# Hepatic Surf4 Deficiency Impairs Serum Amyloid A1 Secretion and Attenuates Liver Fibrosis in Mice

**DOI:** 10.34133/research.0435

**Published:** 2024-08-05

**Authors:** Bingxiang Wang, Huili Li, Govind Gill, Xiangyan Zhang, Geru Tao, Boyan Liu, Lei Zhai, Wei Chen, Hao Wang, Hong-mei Gu, Shucun Qin, Da-wei Zhang

**Affiliations:** ^1^ School of Clinic and Basic Medical Sciences, The Second Affiliated Hospital of Shandong First Medical University & Shandong Academy of Medical Sciences, Jinan, China.; ^2^ Institute of Atherosclerosis in Shandong First Medical University & Shandong Academy of Medical Sciences, Taian, China.; ^3^ Department of Pediatrics and Group on the Molecular and Cell Biology of Lipids, Faculty of Medicine and Dentistry, University of Alberta, Edmonton, Alberta, Canada.; ^4^ Department of Pathology, The Affiliated Hospital of Qingdao University, Qingdao, China.

## Abstract

Liver fibrosis is a severe global health problem. However, no effective antifibrotic drugs have been approved. Surf4 is primarily located in the endoplasmic reticulum (ER) and mediates the transport of secreted proteins from the ER to the Golgi apparatus. Knockout of hepatic Surf4 (*Surf4*^LKO^) in mice impairs very-low-density lipoprotein secretion without causing overt liver damage. Here, we found that collagen levels are significantly reduced in the liver of *Surf4*^LKO^ mice compared with control *Surf4*^flox^ mice, as demonstrated by proteomics, Western blot, and quantitative reverse transcription polymerase chain reaction. Therefore, this study aims to investigate whether and how hepatic Surf4 affects liver fibrosis. We observed that CCl_4_-induced liver fibrosis is significantly lower in *Surf4*^LKO^ mice than in *Surf4*^flox^ mice. Mechanistically, hepatic Surf4 deficiency reduces serum amyloid A1 (SAA1) secretion and hepatic stellate cell (HSC) activation. Surf4 coimmunoprecipitates and colocalizes with SAA1. Lack of hepatic Surf4 significantly reduces SAA1 secretion from hepatocytes, and SAA1 activates cultured human HSCs (LX-2 cells). Conditioned medium (CM) from Surf4-deficient primary hepatocytes activates LX-2 cells to a much lesser extent than CM from *Surf4*^flox^ primary hepatocytes, and this reduced effect is restored by the addition of recombinant SAA1 to CM from Surf4-deficient hepatocytes. Knockdown of SAA1 in primary hepatocytes or TLR2 in LX-2 cells significantly reduces LX-2 activation induced by CM from *Surf4*^flox^ hepatocytes but not from *Surf4*^LKO^ hepatocytes. Furthermore, knockdown of SAA1 significantly ameliorates liver fibrosis in *Surf4*^flox^ mice but does not further reduce liver fibrosis in *Surf4*^LKO^ mice. We also observe substantial expression of Surf4 and SAA1 in human fibrotic livers. Therefore, hepatic Surf4 facilitates SAA1 secretion, activates HSCs, and aggravates liver fibrosis, suggesting that hepatic Surf4 and SAA1 may serve as treatment targets for liver fibrosis.

## Introduction

Various stimuli, including metabolic dysfunction-associated steatotic liver disease (MASLD), toxic chemicals, etc., cause chronic liver damage and activate hepatic stellate cells (HSCs), leading to liver fibrosis characterized by excessive hepatic extracellular matrix (ECM) deposition. Millions of patients with liver fibrosis develop hepatic dysfunction and hepatocellular carcinoma (HCC) every year [1–4]. However, no effective antifibrotic drugs, especially for patients with severe cirrhosis, have been approved. Therefore, identification of new therapeutic targets is urgent.

The etiology of liver fibrosis depends on hepatic injuries; however, myofibroblasts (MFBs), primarily producing fibrillar collagen throughout the course of liver fibrosis, primarily originate from activated HSCs [3,5]. The TGF-β1/SMAD pathway has a major role in activating HSC; however, HSC activation also involves non-SMAD pathways, such as NF-κB, p38, JNK, and PI3K/Akt pathways [3,6–8]. These can activate and transdifferentiate HSCs into MFBs, leading to excessive ECM formation and liver fibrosis.

Serum amyloid A1 (SAA1), an acute-phase response (APR) protein, has a marked role in response to inflammatory stimuli. Emerging evidence implicates its profibrotic role [9–15]. The SAA family includes SAA1, 2, 3, and 4 in mice and SAA1, 2, and 4 in humans. SAAs are poorly water soluble and mainly bind to high-density lipoprotein (HDL) in the blood. SAA4 is constitutively expressed at a low level, while SAA1 and SAA2 are APR proteins. SAA1 and 2 are prologue genes with 93% identical amino acid composition. However, studies indicate that SAA1 is the predominant isoform, accounting for approximately 74% of total SAA in healthy individuals, and has greater pathogenic properties [16,17]. SAA1 is highly conserved among mammals, with 76% of the amino acid residues identical in human and mouse SAA1. SAA1 is present at low levels in healthy individuals but increases dramatically during APR and decreases rapidly after the APR event is resolved. Chronic inflammation also substantially increases hepatic expression and plasma levels of SAA1 [11,13–15,18]. SAA1 can increase hepatic steatosis, activate HSCs, and increase ECM deposition in the liver via the Toll-like receptor (TLR) pathways, and its levels are increased in the liver and blood of patients with MASLD [11,19–21]. SAA1 is expressed in a variety of cells, but hepatocytes contribute the majority of circulating SAA1 under basal and stimulated conditions. How SAA1 is transported from the endoplasmic reticulum (ER) and secreted from cells, however, is still unknown.

Surf4 is a cargo receptor in the ER membrane that facilitates protein secretion [22]. It mediates chylomicron and very low-density lipoprotein (VLDL) secretion from enterocytes and hepatocytes, respectively. However, liver-specific Surf4 knockout (*Surf4*^LKO^) mice do not exhibit substantial hepatic lipid accumulation or liver damage despite impaired VLDL secretion [23,24]. To further understand the physiological role of hepatic Surf4, we performed proteomics analysis and observed markedly lower collagen levels in the liver of *Surf4*^LKO^ mice than that of *Surf4*^flox^ mice. Additionally, CCl_4_ significantly increased plasma SAA1 in *Surf4*^flox^ mice but not in *Surf4*^LKO^ mice. Hepatic Surf4 deficiency significantly decreased SAA1 secretion from isolated primary hepatocytes, lowered circulating SAA1 levels, and attenuated liver fibrosis in mice receiving CCl_4_. Conditioned medium (CM) from primary hepatocytes of *Surf4*^LKO^ mice activated cultured human HSCs, LX-2 cells, to a much lesser extent than CM from primary hepatocytes of *Surf4*^flox^ mice. This reduced effect on LX-2 activation was restored when recombinant SAA1 was added to the CM of Surf4-deficient primary hepatocytes. Furthermore, knockdown of SAA1 significantly reduced liver fibrosis in *Surf4*^flox^ mice but did not further reduce liver fibrosis in *Surf4*^LKO^ mice. Therefore, Surf4 mediates hepatic SAA1 secretion, which activates HSCs and exacerbates liver fibrosis.

## Results

### Liver gene expression impacted by Surf4 deficiency

**Fig. 1.**
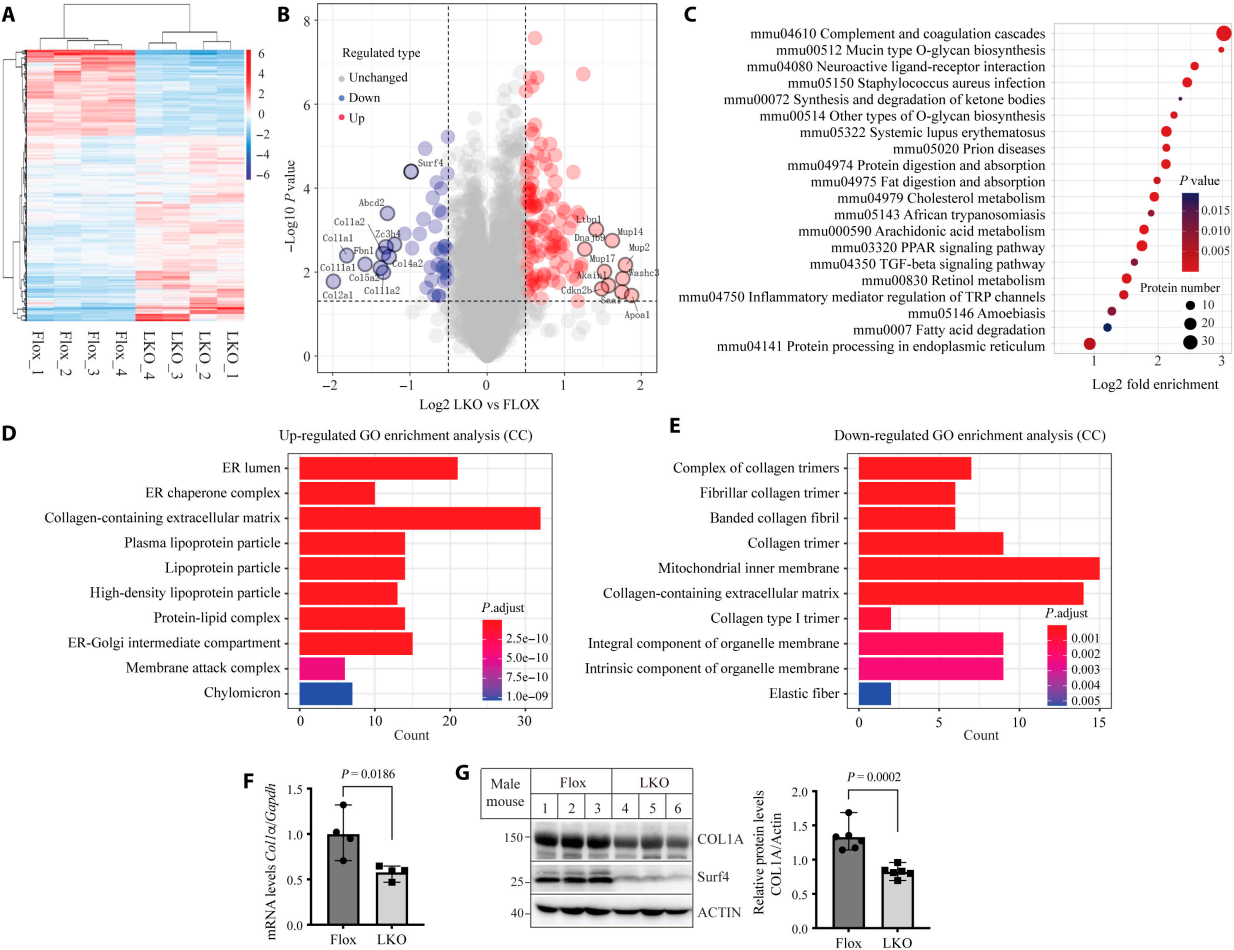
Gene expression in the liver of *Surf4*^LKO^ mice. (A to E) Proteomics of livers of *Surf4*^flox^ and *Surf4*^LKO^ mice (male, *n* = 4 mice). Heat map (A), volcano plot (B), KEGG pathway enrichment (C), and GO function enrichment analysis of up-regulated (D) and down-regulated proteins (E). Heat map and volcano plot showed differentially expressed genes between *Surf4*^flox^ and *Surf4*^LKO^ mice. In heat maps, each line represents a protein, and each column represents a mouse. Bioinformation analysis diagrams were generated by the Hiplot Pro Biomedical visualization platform of the Shanghai Tengyun cloud mapping system. (F and G) Expression of COL1A. Total RNA and tissue homogenate from the liver of male *Surf4*^flox^ and *Surf4*^LKO^ mice for qRT-PCR (F, 4 mice per group) and immunoblotting (G, 6 mice per group, representative images), respectively. The ratio of the mRNA levels of *Col1a* to *Gapdh* or densitometry of COL1A to actin was used as relative expression.

Proteomics analysis of the liver of *Surf4*^flox^ and *Surf4*^LKO^ mice revealed 5,681 quantifiable proteins (330 up-regulated and 153 down-regulated) between the 2 genotypes (Fig. 1A and B and Table S1). Bioinformatic enrichment analysis revealed multiple altered pathways involved in lipid metabolism, for example, synthesis and degradation of ketone bodies, fat digestion and absorption, cholesterol metabolism, fatty acid degradation, retinol metabolism, and peroxisome proliferator-activated receptor signaling pathway. Various other pathways, such as complement and coagulation cascades, protein metabolism, inflammatory mediator regulation of TRP channels, and TGF-β signaling pathway, were also enriched in proteins with altered expression (Fig. 1C). These findings indicate that hepatic Surf4 deficiency alters diverse biological processes in mouse liver.

Cellular component (CC) enrichment analysis of 330 up-regulated proteins revealed protein enrichment on lipoprotein particles and protein-lipid particles, as well as ER lumen and ER chaperone complex and ER-Golgi intermediate compartment in the liver of *Surf4*^LKO^ mice (Fig. 1D), consistent with previous reports that Surf4 mediates lipoprotein secretion [22–27]. Interestingly, the pathway enriched with the highest number of proteins with altered expression was the collagen-containing ECM. Many other pathways, such as blood coagulation, endopeptidase inhibitor activity, and peptidase regulator activity, were also revealed with biological process (BP) and molecular function (MF) analysis (Fig. S1A and B). The top 5 up-regulated proteins included apolipoprotein A-I (APOA-I, Log2Fold change [FC] LKO/Flox = 3.67, *P* = 0.0362), major urinary protein 2 (MUP2, LKO/Flox = 3.47, *P* = 0.0068), WASH complex subunit 3 (WASHC3, Log2FC LKO/Flox = 3.387, *P* = 0.0144), SAA1 (Log2FC LKO/Flox = 3.359, *P* = 0.0299), and MUP14 (Log2FC LKO/Flox = 3.079, *P* = 0.0018) (Table S1). APOA-I is the main structural apolipoprotein of HDL. Lack of Surf4 may directly or indirectly affect APOA-I secretion, leading to extremely low plasma HDL levels in *Surf4*^LKO^ mice [24]. MUPs are secretory proteins expressed in mice but not in humans. Their physiological functions remain elusive. Mice deficient in MUPs are indistinguishable from wild-type littermates [28]. WASHC3, an intracellular protein primarily located in the endosome membrane, regulates endosomal F-actin polymerization, endosomal sorting, and exocytosis [29]. SAA1 is a secretory protein and is involved in diverse roles, such as APR, tissue amyloid deposits, and lipid metabolism [28]. Surf4 mediates protein ER-Golgi export; therefore, the change in these proteins may be caused directly or indirectly by Surf4 deficiency. The distinct functions of these proteins further indicate the complex changes in the liver caused by lacking hepatic Surf4.

We then analyzed the 153 down-regulated proteins with the Gene Ontology (GO) enrichment and also observed changes in multiple pathways. Fatty acid metabolic process and biosynthetic process were enriched in BP analysis (Fig. S1C), consistent with our previous report that hepatic Surf4 deficiency lowers the expression of de novo lipogenesis genes [24,25]. Interestingly, collagen and ECM pathways were also significantly enriched in the CC, BP, and MF analyses (Fig. 1E and Fig. S1C and D). The top 8 most significantly down-regulated proteins were ECM components, including COL3A1, COL2A1, COL1A1, COL11A1, COL5A2, COL1A2, COL11A2, and Fibrillin 1 (FBN1) (Fig. 1B and Table S1). Data in Fig. 1F and G confirmed a significant reduction in mRNA and protein levels of COL1A in the liver of *Surf4*^LKO^ mice, indicating that Surf4 deficiency reduces hepatic collagen expression.

### Impact of Surf4 deficiency on plasmid lipids

**Fig. 2.**
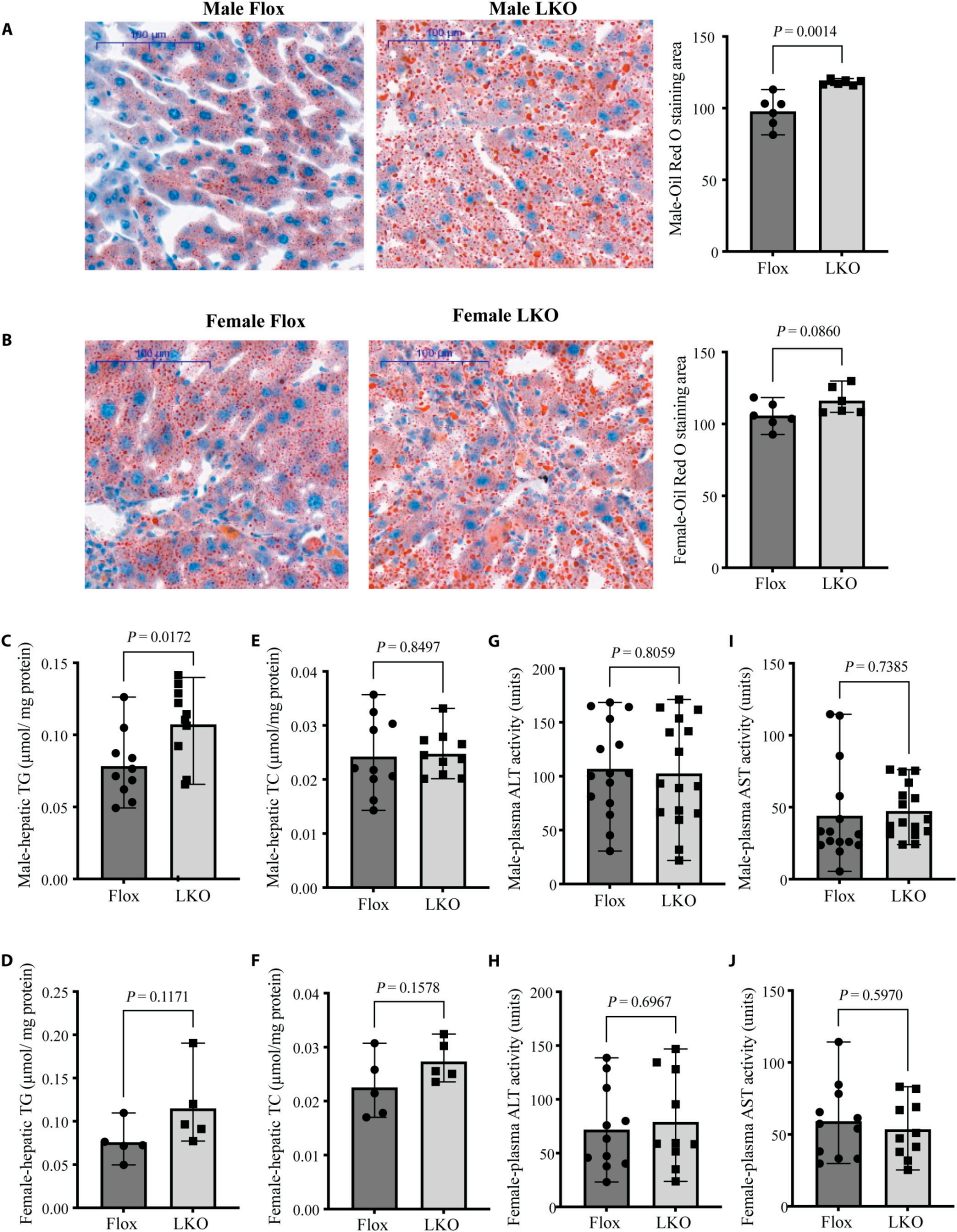
Hepatic lipid levels and liver function of mice administered CCl_4_. Male and female *Surf4*^flox^ and *Surf4*^LKO^ mice (10 to 12 weeks old) fed a regular chow diet were administered CCl_4_ for 6 weeks. Mice were euthanized after 10 h of fasting. (A and B) Oil Red O staining (*n* = 6 mice, representative images). Liver sections were subjected to staining and then quantified using ImageJ. (C to F) Hepatic lipids normalized to total protein concentrations. TG (C and D) and TC (E and F) (*n* = 10 mice). (G to J) Plasma ALT (G and H) and AST (I and J) activity (14 to 16 mice per group).

Excessive accumulation of ECM leads to liver fibrosis. Therefore, we investigated whether the substantial reduction in ECM in *Surf4*^LKO^ mice affects liver fibrosis. Mice fed a high fat for 50 weeks developed hepatic steatosis with little fibrosis. A methionine- and choline-deficient (MCD) diet (high in sucrose [40%] and fat [10%] but deficient in methionine and choline) can induce notable liver fibrosis after approximately 20 weeks of feeding. However, the MCD diet results in marked weight loss, which is uncommon in patients with metabolic syndromes. On the other hand, CCl_4_ can induce liver fibrosis quickly. This is the most commonly used mouse model of liver fibrosis and resembles many aspects of human liver fibrosis caused by chronic disease [30–32]. Therefore, we administered mice with CCl_4_ for 6 weeks. Plasma TG, total cholesterol (TC), HDL-cholesterol and non-HDL-cholesterol levels were significantly lower in both male and female *Surf4*^LKO^ mice than those in *Surf4*^flox^ mice (Fig. S2A to H), consistent with our previous reports [24,25,33]. On the other hand, Surf4 knockout had no significant effect on blood glucose levels or body weight in male or female mice (Fig. S3A to D). Liver weight was increased in male and female *Surf4*^LKO^ mice. However, the liver/body weight ratio, lipid droplets, and TG levels showed a mild but statistically significant increase only in male *Surf4*^LKO^ mice (Fig. S3E to H and Fig. 2A and C). Female *Surf4*^LKO^ mice also exhibited an increasing trend in these parameters, but the difference did not reach statistical significance (Fig. 2B and D). However, hepatic TC levels and plasma alanine transaminase (ALT) and aspartate aminotransferase (AST) activity were comparable between the 2 groups in both male and female mice (Fig. 2E to J). Therefore, hepatic Surf4 silencing results in mild hepatic TG accumulation, especially in male mice, but does not significantly exacerbate liver damage in mice receiving CCl_4_.

### Impact of Surf4 deficiency in liver fibrosis and HSC activation

**Fig. 3.**
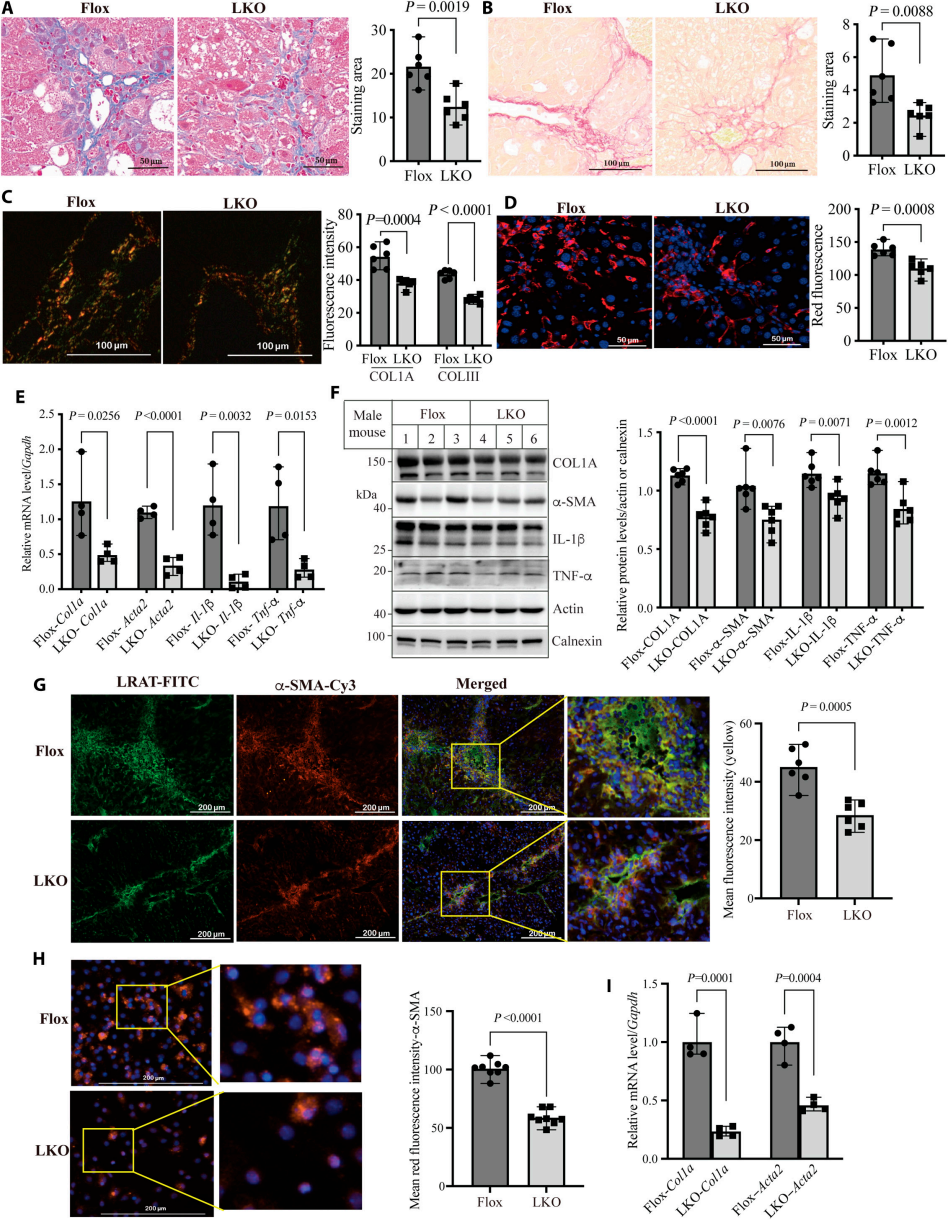
Liver fibrosis in male *Surf4*^flox^ and *Surf4*^LKO^ mice. Male *Surf4*^flox^ and *Surf4*^LKO^ mice (10 to 12 weeks old, regular diet) were administered CCl_4_ for 6 weeks and then euthanized after 10 h of fasting. (A to C) Histology of liver sections. Masson’s (A) or Sirius red staining (B) followed by polarized light microscopy (C) to quantify collagen content (red and green indicated COLI and III, respectively) (6 mice per group). (D) Immunostaining of liver sections with F4/80 (Red, *n* = 6 mice). (E and F) Gene expression. qRT-PCR of liver samples of *Surf4*^flox^ and *Surf4*^LKO^ mice (E, *n* = 4 mice). The mRNA levels of target genes were normalized to that of *Gapdh* for calculating relative mRNA levels. The same amount of liver homogenate was applied to Western blot (F, *n* = 6 mice). The densitometry of COL1A, IL-1β, or TNF-α was normalized to that of actin on the same blot for relative expression. α-SMA was normalized to calnexin. (G and H) Immunohistochemistry. Liver sections (G, 6 mice per group) or primary HSCs (H, 8 mice per group) were incubated with an anti-α-SMA antibody (red). Liver sections were also blotted with an anti-LRAT antibody (green). DAPI: blue. Antibody binding was detected using immunofluorescence microscopy. (I) mRNA levels of *Col1α* and *Acta2* measured by qRT-PCR (4 mice per group). (A to D and F to H) Representative images were shown. Data were quantified using ImageJ.

Masson’s trichrome and Sirius red staining of liver sections showed reduced fibrosis in male and female *Surf4*^LKO^ mice compared with their control (Fig. 3A and B and Fig. S4A and 4B). Detailed analysis of Sirius red-staining sections using polarized light microscopy consistently revealed a significant reduction in collagen content in *Surf4*^LKO^ mice (Fig. 3C and Fig. S4C). Furthermore, immunostaining of liver sections with F4/80, a macrophage marker, showed that Surf4 deficiency significantly reduced macrophage populations (Fig. 3D and Fig. S4D). Lack of Surf4 also significantly reduced the expression of liver fibrosis markers (*Col1a*, *Acta2/α-Sma*) and inflammation markers (*Il-1β* and *Tnf-α*) at mRNA and protein levels in male and female *Surf4*^LKO^ mice (Fig. 3E and F and Fig. S4E and F). We noticed a statistically significant but mild decrease in the protein levels of COL1A, α-SMA, TNF-α, and IL-1β in the liver of *Surf4*^LKO^ mice (Fig. 3F and Fig. S4F). Notably, HSCs constitute only about 5% to 10% of hepatocytes but are responsible for phenotypic changes in the liver. Whole liver homogenate was used for Western blotting in Fig. 3, which may be attributed to the mild effect observed. Nevertheless, knockout of hepatic Surf4 reduces collagen content and inflammation in the liver of mice receiving CCl_4_, thereby attenuating liver fibrosis.

HSC activation is the primary driver of ECM production and liver fibrosis and has been implicated as the exclusive source of the MFB pool in CCl_4_-induced liver fibrosis [34]. Therefore, to understand how knockout of hepatic Surf4 affected CCl_4_-induced liver fibrosis, we evaluated expression of lecithin:retinol acyltransferase (LRAT), an HSC cell marker, and α-SMA, a marker for HSC activation. As shown in Fig. 3G, both LRAT and α-SMA staining and their co-staining in liver sections were significantly lower in *Surf4*^LKO^ mice than in *Surf4*^flox^ mice, indicating fewer total and activated HSCs. Consistently, α-SMA positive staining and its mRNA levels (Fig. 3H and I) and the protein levels of α-SMA, COL1A, and TNF-α (Fig. S4G) were significantly reduced in primary HSCs isolated from *Surf4*^LKO^ mice, indicating that hepatic Surf4 deficiency suppresses HSC activation in CCl_4_-treated mice.

### Plasma proteins and SAA1 secretion in *Surf4*^LKO^ mice

**Fig. 4.**
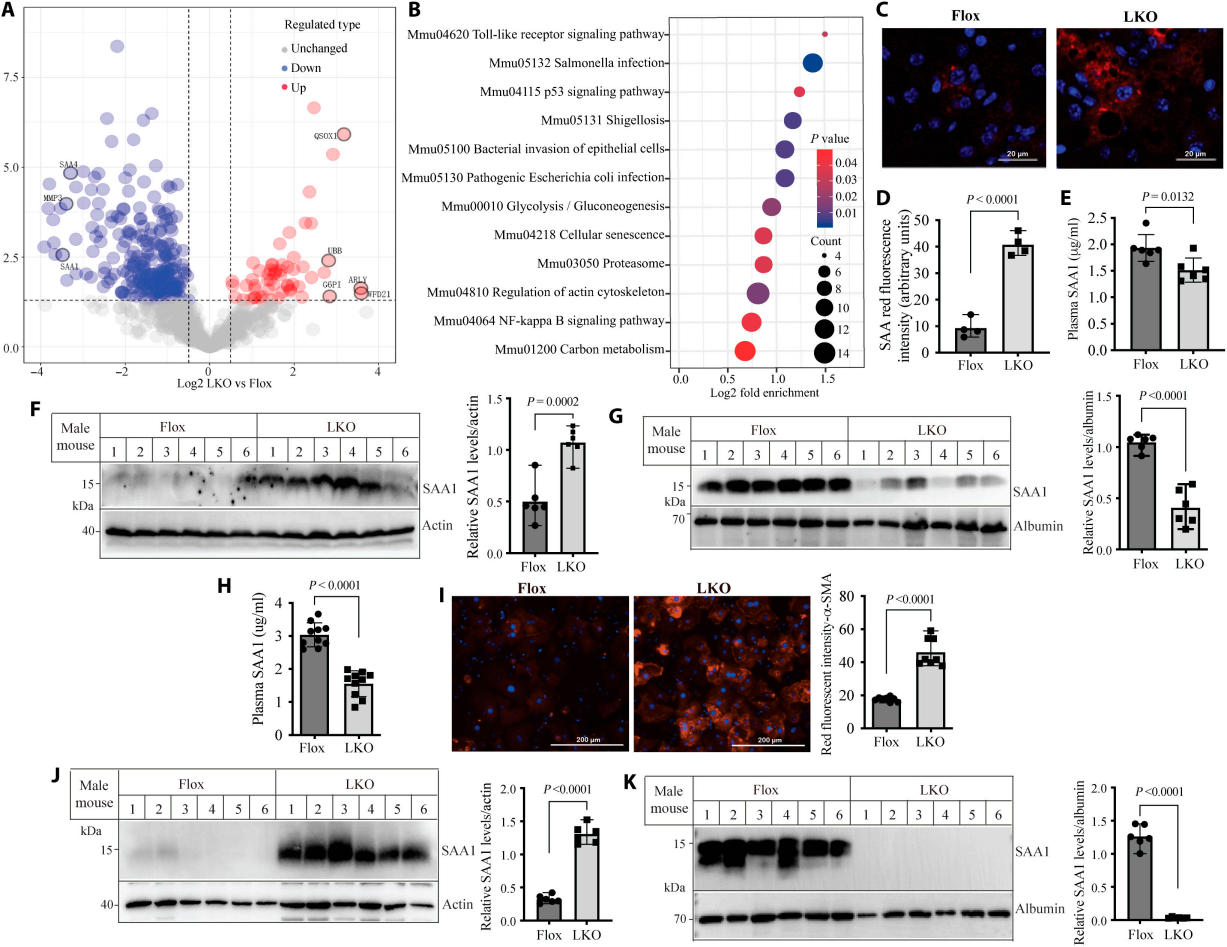
Impact of hepatic Surf4 knockout on plasma protein and hepatic SAA1 secretion. (A and B) Proteomics of plasma of different male mice (*n* = 5), including volcano plot (A) and KEGG analysis (B). (C to G) SAA1 levels in mice without CCl_4_ treatment (male, regular diet, 10 to 14 weeks old). (C and D) Immunofluorescence of liver samples of male *Surf4*^flox^ and *Surf4*^LKO^ mice (*n* = 4). SAA1: red. DAPI: blue. Data were quantified using ImageJ. Fasting plasma SAA1 levels were assessed using ELISA (E, *n* = 6 male mice). Immunoblotting of an equal amount of whole cell lysate (F) or culture medium (G) of primary hepatocytes isolated from different male *Surf4*^flox^ and *Surf4*^LKO^ mice (10 to 12 weeks old, *n* = 6 mice). (H and I) SAA1 levels in mice receiving CCl_4_ for 6 weeks (male, regular diet, 10 to 14 weeks old). Fasting plasma levels of SAA1 were measured using ELISA (H, *n* = 10 mice). Primary hepatocytes from different male mice (*n* = 8) were applied to immunofluorescence (I, SAA1: red, representative images). (J and K) Immunoblotting of SAA1 in cell lysate (J) and culture medium (K) of primary hepatocytes from different mice (*n* = 6 mice). The densitometry of SAA1 was normalized to that of actin or albumin for relative levels. Data were quantified using ImageJ. Statistics: Unpaired Student’s *t* test.

TGF-β is critical for HSC activation. However, unlike HSCs, primary hepatocytes of *Surf4*^flox^ and *Surf4*^LKO^ mice displayed virtually undetectable expression of TGF-β (Fig. S5A and B), consistent with previous reports [35–38]. To understand how knockout of Surf4 in hepatocytes affected HSC activation and liver fibrosis, we performed proteomics on plasma samples and identified 932 proteins (57 up-regulated and 190 down-regulated) in *Surf4*^LKO^ mice compared with *Surf4*^flox^ mice (Fig. 4A, Fig. S5C, and Table S2). Plasma proteins with significantly altered expression were involved in diverse processes, such as carbohydrate transport and metabolism, signaling transduction mechanisms and posttranslational modifications, protein turnover, and chaperons (Fig. S5D). Kyoto Encyclopedia of Genes and Genomes (KEGG) analysis showed that multiple pathways were enriched with proteins with the most significant changes in expression, such as the TLR and NF-kappa B pathway, and Salmonella infection (Fig. 4B). Consistently, GO analysis of up-regulated proteins revealed the involvement of diverse pathways, such as the small-molecule catabolic process and reactive oxygen species metabolic process in BP, carbohydrate binding and antioxidant activity in MF, and collagen-containing ECM and myelin sheath in CC (Fig. S6A to C). In addition, GO analysis of down-regulated proteins showed that several BPs and CCs involved in lipid metabolism (e.g., lipid localization, protein-lipid complex remodeling, and various plasma lipoprotein particles) were enriched in *Surf4*^LKO^ mice (Fig. S6D to F), consistent with the role of Surf4 in mediating lipoprotein secretion [24,25,33]. However, down-regulated proteins also participated in many other cellular functions and processes, such as negative regulation of hydrolase activity in BP, glycosaminoglycan binding in MF, and collagen-containing ECM in CC. Therefore, lacking hepatic Surf4 causes changes in plasma proteins involved in diverse functions.

Many significantly down-regulated proteins identified in proteomics of plasma samples were apolipoproteins and Ig chains (Table S2). We noticed that plasma SAA1 levels were also remarkably reduced in *Surf4*^LKO^ mice (Log2FC LKO/Flox = −3.16, *P* = 0.009, Table S2 and Fig. 4A), but significantly increased in the liver of *Surf4*^LKO^ mice (Log2FC LKO/Flox = 3.358, *P* = 0.0299, Table S1). SAA1 produced from hepatocytes has been reported to activate HSCs locally and promote hepatic ECM deposition in mice administered a single dose of 20% CCl_4_ [11]. Therefore, we investigated the possibility that Surf4-deficient hepatocytes impaired SAA1 secretion and consequently reduced HSC activation and liver fibrosis. Immunohistochemistry showed accumulation of SAA1 in the liver and primary hepatocytes of *Surf4*^LKO^ mice (Fig. 4C and D and Fig. S7A). We also observed overlap of SAA1 with an ER-tracker (orange puncta, Fig. S7B), indicating ER accumulation of SAA1 in Surf4-deficient hepatocytes. We then measured plasma SAA1 levels and observed a significant reduction in *Surf4*^LKO^ mice (Fig. 4E). Circulating SAA1 is also secreted from extrahepatic tissues, e.g., adipose tissue, the intestine, etc. [14], which may contribute to plasma SAA1 in *Surf4*^LKO^ mice.

We further isolated primary hepatocytes and measured SAA1 secretion. SAA1 was significantly elevated in cell lysate but decreased in culture medium of primary hepatocytes isolated from *Surf4*^LKO^ mice (Fig. 4F and G). We then evaluated changes in SAA1 in mice receiving CCl_4_ for 6 weeks. Surf4 deficiency significantly reduced plasma SAA1 levels and caused accumulation of SAA1 in the liver of *Surf4*^LKO^ mice (Fig. 4H and I). Consistently, Surf4-deficient primary hepatocytes exhibited a significant increase in SAA1 levels in whole cell lysate and a significant decrease in secreted SAA1 in culture medium (Fig. 4J and K). Furthermore, CCl_4_ significantly increased plasma SAA1 levels in *Surf4*^flox^ mice (mean: 3.03 in CCl_4_ treatment [Fig. 4H] and 1.93 in no CCl_4_ treatment [Fig. 4E], *P* < 0.0001), but not in *Surf4*^LKO^ mice (mean: 1.56 in CCl_4_ treatment [Fig. 4H] and 1.51 in no CCl_4_ treatment [Fig. 4E], *P* = 0.8139). Therefore, Surf4 deficiency impairs SAA1 secretion from hepatocytes.

### The role of SAA1 in HSC activation

**Fig. 5.**
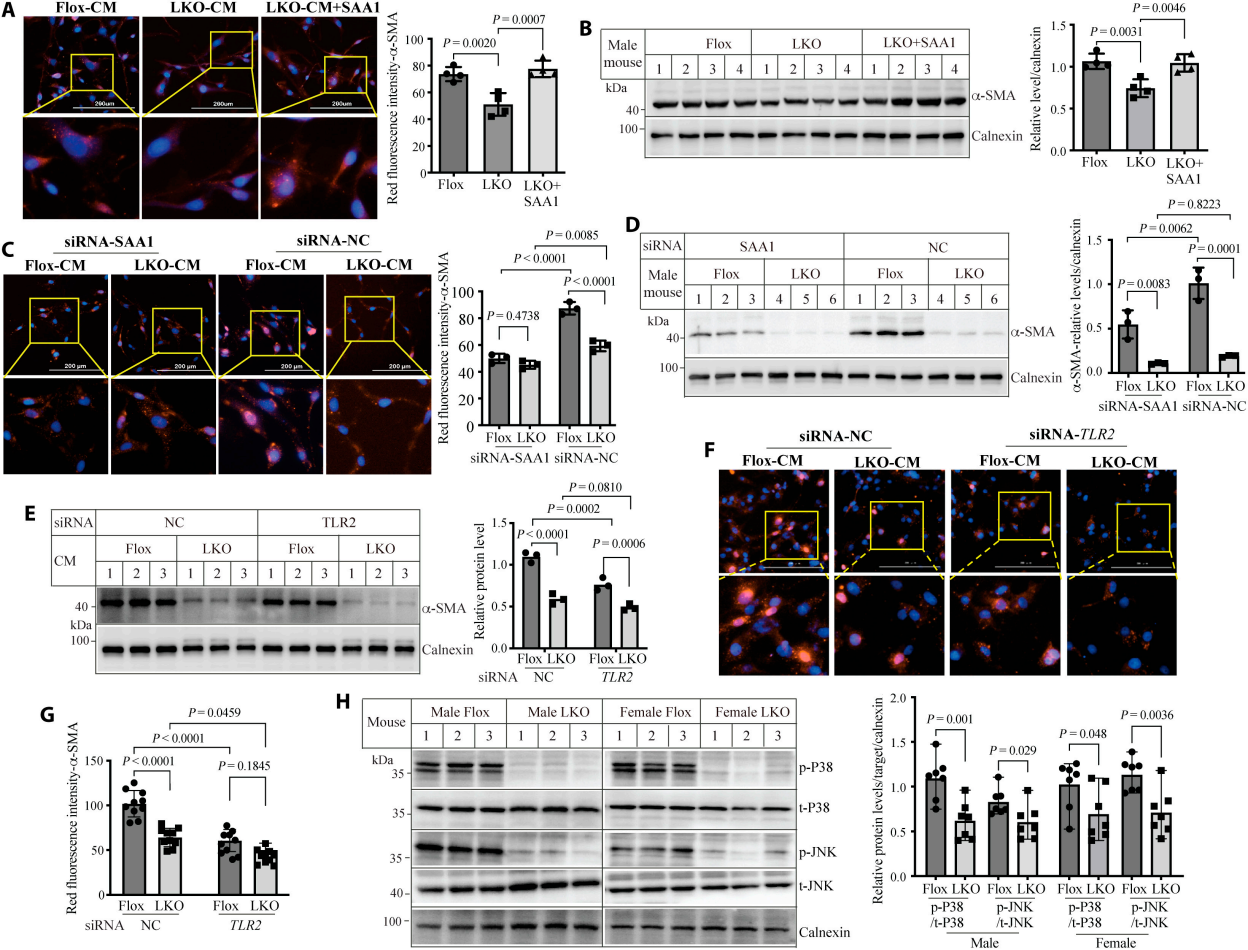
HSC activation affected by SAA1. (A and B) Effect of CM on LX-2 cells. Primary hepatocytes isolated from different male *Surf4*^flox^ and *Surf4*^LKO^ mice (10 to 14 weeks old, *n* = 4) were cultured in medium without FBS. Twenty-four hours later, CM was harvested. LX-2 cells were incubated with CM with or without SAA1 (2.5 mg/ml) for 24 h, followed by immunofluorescence (A, red) or immunoblotting (B). (C and D) SAA1 knockdown. Primary hepatocytes were isolated from different male *Surf4*^flox^ and *Surf4*^LKO^ mice administered CCl_4_ (*n* = 3 mice) and then transfected with negative control (NC) or SAA1 siRNA. CM was then collected. LX-2 cells were incubated with CM for 24 h, followed by immunofluorescence (C, red) or immunoblotting (D). (E to G) α-SMA expression. Twenty-four hours after transfection with negative control (NC) or *TLR2* siRNA, LX-2 cells were incubated with CM collected from primary hepatocytes of different male *Surf4*^flox^ or *Surf4*^LKO^ mice administered CCl_4_. α-SMA was detected by Western blot (E, *n* = 3 biological replicates) or immunofluorescence (F and G, *n* = 10 biological replicates). (H) Immunoblotting. An equal amount of liver homogenate from male and female *Surf4*^flox^ or *Surf4*^LKO^ mice (regular diet) receiving CCl_4_ for 6 weeks was applied to immunoblotting. A relative protein level was calculated by normalizing densitometry of phosphorylated protein to that of the total protein on the same blot. Statistics: A and B, one-way ANOVA; C to G, 2-way ANOVA; H, Student’s *t* test.

Liver fibrogenesis is a complex process, in which HSC activation plays an essential role. Therefore, we assessed a potential direct effect of Surf4 deficiency in hepatocytes on HSC activation. We collected CM from primary hepatocytes of *Surf4*^flox^ and *Surf4*^LKO^ mice to treat cultured human HSCs, LX-2 cells. α-SMA staining and its protein levels were significantly lower in LX-2 cells incubated with CM of *Surf4*^LKO^ hepatocytes compared with CM of *Surf4*^flox^ hepatocytes, and the reduced effect on α-SMA was restored by supplementing CM of *Surf4*^LKO^ hepatocytes with recombinant SAA1 (Fig. 5A and B). We then isolated primary hepatocytes from CCl_4_-treated *Surf4*^flox^ and *Surf4*^LKO^ mice and transfected them with either negative control (NC) or SAA1 siRNA. SAA1 expression in primary hepatocytes and culture medium was effectively reduced by its siRNA (Fig. S8A). CM was then collected to treat LX-2 cells. Compared with NC siRNA-transfected hepatocytes, knockdown of SAA1 in *Surf4*^flox^ hepatocytes markedly reduced α-SMA staining in LX-2 cells that received CM of *Surf4*^flox^ hepatocytes (Fig. 5C, column 1 vs. 3, mean difference: 37.52, *p* < 0.0001), but the reduction in α-SMA staining was much less in LX-2 cells treated with CM of SAA1-knockdown *Surf4*^LKO^ hepatocytes (column 2 vs. 4, mean difference: 14.1, *P* = 0.0085). Immunoblotting of whole cell lysate with α-SMA showed a similar phenotype (Fig. 5D), with knockdown of SAA1 in *Surf4*^flox^ hepatocytes causing a more significant reduction in α-SMA levels in LX-2 cells (column 1 vs. 3, mean difference: 0.82, *P* = 0.0062) than knockdown of SAA1 in *Surf4*^LKO^ hepatocytes (column 2 vs. 4, mean difference: 0.08, *P* = 0.8223).

We also silenced *Surf4* in the cultured human hepatoblastoma-derived cell line, HepG2 [39]. Knockdown of Surf4 reduced secretory SAA1 in medium and increased SAA1 in whole cell lysate (Fig. S8B and C). CM from Surf4 knockdown HepG2 cells significantly reduced expression and staining of α-SMA in LX-2 cells, which was restored by exogenous addition of recombinant SAA1 (Fig. S8D and E). We then investigated the interaction between SAA1 and Surf4. The interaction between cargo receptors and their substrates is usually weak and transient [40]. Therefore, we co-expressed SAA1 and Surf4 in HepG2 cells and then treated cells with sodium azide and D-glucose to inhibit disassociation between cargo receptors and their substrates, followed by crosslinking with a membrane-permeable reversible crosslinker, as previously described [41]. Surf4 was immunoprecipitated from Surf4-expressing cell lysate (Fig. S8F, lanes 6 and 8). SAA1 was co-immunoprecipitated with Surf4 from lysate of cells co-transfected with Surf4 and SAA1 (lane 8). We also conducted confocal microscopy of HepG2 cells transiently co-transfected with Surf4 and SAA1 and treated with sodium azide and D-glucose (Fig. S8G). Both Surf4 (green fluorescence) and SAA1 (red fluorescence) were detected in transfected cells and showed partial colocalization in the overlapped image (yellow), indicating an association between Surf4 and SAA1. Taken together, these findings indicate that Surf4 mediates SAA1 secretion, which contributes, at least in part, to the effect of Surf4 deficiency on HSC activation.

It has been reported that TLR2 mediates SAA1-induced activation of HSCs (11). Proteomics analysis of plasma samples revealed enrichment in the TLR signaling pathway (Fig. 4B). Therefore, we knocked down TLR2 expression in LX-2 cells using siRNA. *TLR2* siRNA significantly reduced *TLR2* mRNA level compared with control siRNA (Fig. S8H). Cells were then incubated with CM collected from *Surf4*^flox^ and *Surf4*^LKO^ hepatocytes. Compared with LX-2 cells transfected with control siRNA, LX-2 cells transfected with *TLR2* siRNA displayed a marked decrease in α-SMA levels when treated with CM of *Surf4*^flox^ hepatocytes (Fig. 5E, column 3 vs. 1, mean difference: 0.51, *P* = 0.0002), but showed a much less reduction in α-SMA when treated with CM of *Surf4*^LKO^ hepatocytes (Fig. 5E, column 4 vs. 2, mean difference: 0.1, *P* = 0.081). A consistent phenotype was observed in immunofluorescence, and knockdown of *TLR2* caused a greater reduction in α-SMA staining in LX-2 cells incubated with CM from *Surf4*^flox^ hepatocytes (Fig. 5F and G, column 3 vs. 1, mean difference=41.07, *P* < 0.0001) than that in cells incubated with CM from *Surf4*^LKO^ hepatocytes (Fig. 5F and G, column 4 vs. 2, mean difference: 14.14, *P* = 0.0459). In HSCs, TLRs-MyD88 activates downstream signaling molecules, such as P38 and JNK, promoting liver fibrosis. As shown in Fig. 5H, phosphorylated P38 (p-P38) and phosphorylated JNK (p-JNK) were significantly down-regulated in the liver of male and female *Surf4*^LKO^ mice receiving CCl_4_ compared with *Surf4*^flox^ mice administered CCl_4_, whereas the levels of total P38 and JNK were comparable in the 2 groups. Therefore, TLR2 may mediate the effect of SAA1 on HSC activation.

### The effect of SAA1 knockdown on liver fibrosis

**Fig. 6.**
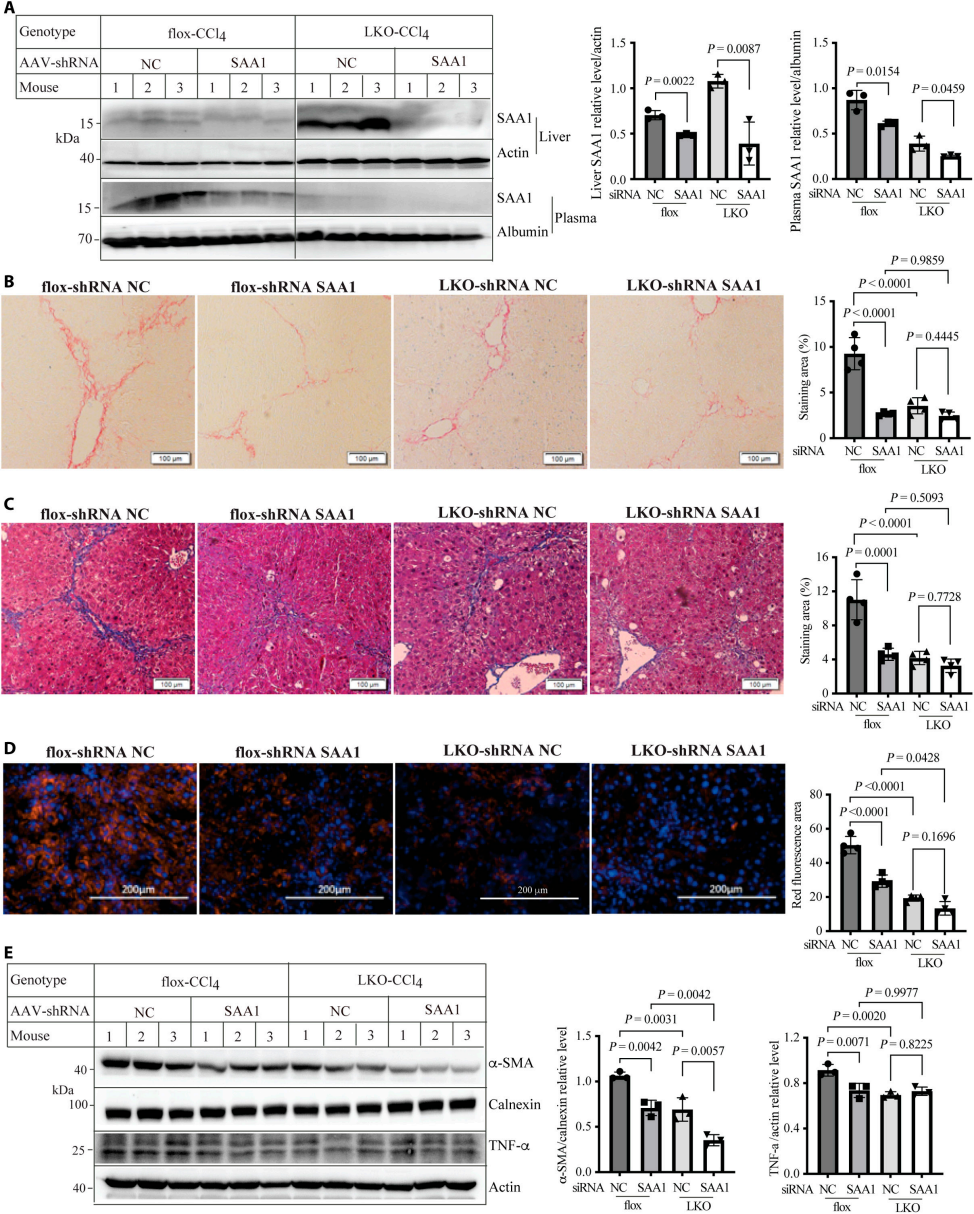
SAA1 knockdown and liver fibrosis. *Surf4*^flox^ and *Surf4*^LKO^ mice (male, 10 to 12 weeks old, regular diet) were injected with AAV-negative control (NC) or SAA1 shRNA, followed by administration of CCl_4_ for 4 weeks. Mice were fasted and euthanized for analysis. (A) SAA1 levels (*n* = 3). SAA1 in liver homogenate and plasma was assessed using Western blot. Relative protein levels were calculated by normalizing the densitometry of SAA1 to that of loading control on the same membrane. Student’s *t* test. (B to D) Histology and immunostaining (*n* = 4). Liver sections were subjected to Sirius red staining (B), Masson’s (C), or immunostaining with α-SMA (D, red). Representative images were shown, and data were quantified using ImageJ. (E) Immunoblotting of liver homogenate (*n* = 3 mice). The same amount of homogenate was used for Western blot. A relative protein level was calculated by normalizing α-SMA densitometry to calnexin densitometry or TNF-α densitometry to actin densitometry on the same membrane. B to E, 2-way ANOVA.

We then investigated whether SAA1 affected liver fibrosis in mice. AAV-shRNA was used to knock down SAA1 in *Surf4*^flox^ and *Surf4*^LKO^ mice. AAV-shRNA SAA1 effectively reduced SAA1 levels in liver homogenate and plasma in *Surf4*^flox^ and *Surf4*^LKO^ mice compared to mice injected with AAV-shRNA NC (Fig. 6A). As shown in Fig. 6B and C, knockdown of SAA1 significantly decreased liver fibrosis in *Surf4*^flox^ mice but not in *Surf4*^LKO^ mice compared with corresponding controls receiving AAV-shRNA NC, as evidenced by Masson’s and Picrosirius red staining (column 1 vs. 2 and column 3 vs. 4). Masson’s and Sirius red staining was also significantly lower in *Surf4*^LKO^ mice receiving AAV-shRNA NC than *Surf4*^flox^ mice receiving AAV-shRNA NC (column 3 vs. 1). However, *Surf4*^flox^ mice and *Surf4*^LKO^ mice showed comparable staining when SAA1 was knocked down (column 2 vs. 4). In addition, immunostaining of liver sections with α-SMA showed that SAA1 knockdown markedly reduced α-SMA levels in *Surf4*^flox^ mice but not *Surf4*^LKO^ mice compared with respective control mice receiving AAV-shRNA NC (Fig. 6D). Knockdown of SAA1 also significantly lowered α-SMA and TNF-α protein levels in *Surf4*^flox^ mice (Fig. 6E). Collectively, these findings indicate that knockdown of SAA1 markedly attenuates liver fibrosis in *Surf4*^flox^ mice but not in *Surf4*^LKO^ mice.

We also collected liver samples from patients with HCC. The para-HCC region displayed high fibrosis, evidenced by Masson’s and Picrosirius red staining (Fig. S9A and B). IHC analyses showed substantial expression of Surf4 and SAA1 in the liver samples (Fig. S9C and D). Altogether, these data suggest that Surf4 mediates SAA1 secretion, which may activate HSCs in a paracrine manner via TLR2, thus exacerbating liver fibrosis (Fig. S9E).

## Discussion

We previously reported that knockout of hepatic Surf4 causes a similar phenotype in male and female mice, dramatically reducing VLDL secretion and plasma TG and cholesterol levels [23–25,33]. On the other hand, knockout of intestinal Surf4 leads to more dramatic death in male mice than in female mice, although intestinal chylomicron secretion is markedly reduced in male and female mice [23]. Here, we observed that hepatic Surf4 deficiency has a similar effect on plasma lipids and liver fibrosis in male and female mice administered CCl_4_, implying no notable impact of sex differences. We previously reported that loss of hepatic Surf4 does not lead to detectable liver damage or hepatic TG accumulation in mice [24,25,33], even though the underlying mechanism remains unclear. Conversely, *Surf4*^LKO^ mice display a mild increase in hepatic TG levels after CCl_4_ treatment. CCl_4_ can increase fatty acid and TG synthesis in hepatocytes [42], which, in combination with impaired VLDL secretion, may lead to increased hepatic TG levels in *Surf4*^LKO^ mice. However, hepatic Surf4 deficiency does not worsen liver damage in mice receiving CCl_4_, as supported by no detectable changes in plasma ALT and AST levels.

The activation and transdifferentiation of HSCs into MFBs are essential for the occurrence and progression of liver fibrosis, and TGF-β plays a central role in HSC activation. Nonparenchymal and inflammatory cells, such as Kupffer cells, bone marrow-derived macrophages, and activated HSCs, rather than hepatocytes, are the main origin of TGF-β in liver fibrosis development [35–38]. We also consistently observed extremely low expression of TGF-β in hepatocytes. Therefore, lack of hepatic Surf4 may not directly affect TGF-β production in the liver. However, TGF-β is activated by multiple factors, such as inflammation and changes in ECM components and stiffness*. Surf4*^LKO^ mice exhibit decreased inflammatory factors and ECM deposition, which might reduce TGF-β production and activation and thus contribute to attenuation of liver fibrosis.

SAA1 has been reported to promote renal interstitial and cardiac fibrosis and activate HSCs [11–15]. Our current findings showed that Surf4 plays an essential role in hepatic SAA1 secretion. Surf4 deficiency markedly reduces SAA1 secretion from hepatocytes isolated from mice treated with or without CCl_4_. Lipid-free SAA in the extracellular milieu can bind to different receptors on the cell surface locally, exerting various physiological and pathophysiological functions [18,43–46]. In acute liver damage caused by CCl_4_ or cryoinjury, hepatocytes produce SAA1, which activates HSCs locally and increases ECM deposition at injury sites [11]. SAA1 deficiency also dramatically reduces cardiac fibrosis in mice [15]. We found that lack of Surf4 markedly reduces HSC activation in CCl_4_-treated mice. In addition, CM of Surf4-deficient primary hepatocytes has a markedly reduced ability to activate LX2 cells compared with CM of primary hepatocytes from *Surf4*^flox^ mice, and this reduction in LX2 activation is restored by supplementing CM of Surf4-deficient primary hepatocytes with recombinant SAA1, indicating the critical role of SAA1 in HSC activation. Furthermore, SAA1 knockdown, like hepatic Surf4 deficiency, substantially reduces liver fibrosis in *Surf4*^flox^ mice, but has no detectable effect on liver fibrosis in *Surf4*^LKO^ mice, suggesting the contribution of SAA1 to Surf4 deficiency-induced alleviation of liver fibrosis in mice. Notably, Surf4 mediates secretion of various cargos [22,47]. Proteomics analysis of liver samples revealed that more than 300 protein levels are increased in *Surf4*^LKO^ mice compared with *Surf4*^flox^ mice. Thus, these changes may also directly or indirectly affect CCl_4_-induced liver fibrosis in mice.

How does SAA1 secreted from hepatocytes activate HSCs? Emerging studies show that TLR2 is a functional receptor for SAA1 [11,48–50]. SAA1 binds TLR2, activates the NF-κB pathway, and increases p-P38 and p-JNK levels in diverse cells, e.g., dermal fibroblasts, macrophages, HeLa cells, and dendritic cells [51–54]. Recently, Getachew et al. reported that SAA1 secreted from hepatocytes binds to TLR2 on HSCs in a paracrine manner, activating and recruiting HSCs to the injury site. Deficiency of SAA1 or TLR2 markedly reduces SAA1-induced HSC activation and ECM deposition at injury sites [11]. Additionally, TLR2 expression is increased in activated HSCs [55,56]. Lack of TLR2 in mice inhibits HSC activation, reduces p-JNK and p-p38, and attenuates liver fibrosis induced by CCl_4_ [57], and SAA1 deficiency substantially reduces p-P38 and p-JNK in the myocardium [15]. Here, we found that knockdown of TLR2 in LX2 cells markedly reduces HSC activation induced by CM of *Surf4*^flox^ primary hepatocytes, but only mildly reduces HSC activation induced by CM of *Surf4*^LKO^ primary hepatocytes. We also observed that levels of p-P38, p-JNK, IL-1β, and TNF-α in the liver were lower in *Surf4*^LKO^ mice than in *Surf4*^flox^ mice. Therefore, our findings implicate that TLR2 on HSCs may mediate SAA1-induced HSC activation. However, additional studies are required to further confirm this possibility. Nevertheless, our findings indicate that hepatic Surf4 mediates SAA1 secretion from hepatocytes, which activates HSCs in a paracrine manner, likely via TLR2. This subsequently increases expression of fibrogenic and inflammatory proteins (e.g., α-SMA and IL-1β), exacerbating liver fibrosis (Fig. S9E).

Targeting the TGF-β pathway can attenuate liver fibrosis in several preclinical animal models [58]; however, the development and translation of TGF-β-based therapies to treat human patients face significant challenges due to the cell-type-dependent pleiotropic roles of TGF-β. Therefore, identification of novel therapeutic targets is in urgent need. Cell–cell communication plays a crucial role in liver fibrogenesis [59], with HSCs acting as a vital signaling hub. Inflammation accompanies and plays a critical role in all stages of liver fibrosis. Inflammation increases SAA1 expression [18], and we observed that CCl_4_ substantially elevates plasma SAA1 levels in *Surf4*^flox^ mice, but not in *Surf4*^LKO^ mice. Specific targeting of hepatocytes has been successfully achieved using GalNAc-conjugated siRNA or antisense oligonucleotides [60,61]. In different mouse models, silencing of hepatic Surf4 dramatically reduces plasma lipid levels and the development of atherosclerosis without causing overt liver damage [24–27]. Collectively, these findings implicate the therapeutic potential of liver Surf4 and SAA1. However, one limitation of the study is that only chemical-induced liver fibrosis was investigated. In addition, Surf4 facilitates ER export of a variety of proteins. We also noticed mild TG accumulation in the liver of *Surf4*^LKO^ mice, which may be caused by impaired VLDL secretion due to Surf4 deficiency. However, we cannot exclude the possibility that accumulated SAA1 in Surf4-deficient hepatocytes is involved in the development of the mild fatty liver. Furthermore, our findings indicate that SAA1 secreted by hepatocytes activates HSCs and promotes liver fibrosis in mice. SAA1 expression is low in mice and humans under normal conditions. Do elevated SAA1 levels induce or promote liver fibrosis in young and/or aged mice with no or only mild insult, such as high-fat or high-fructose feeding? Therefore, caution should be exercised, and more studies on the physiological and pathophysiological roles of Surf4 and SAA1 are warranted.

## Materials and Methods

### Animal models

*Surf4*^LKO^ mice were generated as described. Briefly, the *Surf4*^flox^ mice, in which exon 2 of the *Surf4* gene was flanked with LoxP sites, were bred with albumin (Alb)-Cre mice (The Jackson Laboratory, 003574) to inactivate Surf4 specifically in the liver [24,33]. Mice were maintained in the animal facility at Shandong First Medical University (Taian, China) with free access to a regular diet (Keao Xieli, Beijing, China) and H_2_O in a climate-controlled facility. CCl_4_ (0.6 μl/g body weight) was injected to each mouse (10 to 14 weeks old, 2 to 5 mice per cage) intraperitoneally once every 2 days for 4 or 6 weeks to induce liver fibrosis. Mice in all experiments were fasted for 10 h unless otherwise indicated [25].

### Cell culture and transfection

Primary hepatocytes were prepared as previously described [24]. Primary HSCs were isolated from the liver [62]. Liver was perfused with Hanks’ buffer with pronase (0.4 mg/ml) and collagenase IV (0.5 mg/ml). After centrifugation at 60 × *g*, supernatant was harvested and applied to a density gradient centrifugation. HSCs were collected and cultured in Dulbecco’s Modified Eagle Medium (DMEM) with 10% fetal bovine serum (FBS) for 2 h. After removal of unattached cells, HSCs were cultured in fresh medium for subsequent experiments. HepG2 (National Collection of Authenticated Cell Cultures) and LX-2 (Procell) cells were cultured in DMEM containing 10% FBS and LX-2 special medium (Procell), respectively. siRNA was introduced into cells (primary hepatocytes, LX-2, and HepG2) using Lipofectamine 3000 (Invitrogen). Equal amounts of plasmids containing HA-tagged SAA1 and Myc-DDK-tagged Surf4 were introduced into HepG2 cells using Lipofectamine 3000 as previously described [63].

### Histochemistry

Fresh livers were fixed in 4% paraformaldehyde. H&E, Oil Red O, Masson’s, and Sirius red staining were performed by Servicebio Technology Co., Ltd. (Wuhan, China). All slices were imaged on an Olympus microscope (BX53). Stained areas were quantified with ImageJ (v1.53e).

### Measurement of plasma and liver samples

Fasting blood was collected into EDTA-coated tubes. Plasma was isolated and subjected to analysis of TG, TC, HDL-C, non-HDL-C, ALT, and AST (Nanjing Jiancheng Bioengineering Institute, China) [24]. Plasma IL-1β and TNF-α levels were measured using mouse-specific ELISA assay kits (mlbio, Shanghai, China). The Folch method was used to extract liver lipids for TG and TC measurement with commercial kits (Applygen, China), which was then normalized to the amount of total proteins in the respective sample [24,33].

### qRT-PCR

TRIzol (Invitrogen, 15596018) was used to extract total RNA, which was used for the synthesis of complementary DNA (cDNA) with HiFi Script gDNA Removal RT MaterMix (CWBIO, China, CW2020M). qRT-PCR was performed using TransStart Green qPCR SuperMix (Transgene Biotech, China, AQ101-03). 2^−ΔΔCt^ was used to calculate gene expression, which was normalized to *Gapdh* expression for a relative level (Primers in Table S2).

### Western blot, immunoprecipitation, and immunofluorescence

RIPA buffer (Solarbio) with protease inhibitors (cOmplete Ultra Tablets, Roche) and PMSF was used to lyse tissue and cells. Cell lysate was collected. Cell culture medium was precipitated with trichloroacetic acid (TCA) [64]. An equal amount of total proteins was used for Western blot with antibodies indicated (Table S2) [24,25]. The same amount of lysate from HepG2 cells transfected with different plasmids was applied to anti-Myc antibody-conjugated beads (Sigma) to pull down Myc-tagged Surf4, which was then detected by immunoblotting [65].

Immunofluorescence was performed as in Gu et al. [66]. After fixation, permeabilization, and blocking in 5% bovine serum albumin, cells were incubated with a primary antibody and then a secondary antibody. Nuclei and ER were revealed with DAPI and an ER tracker. Cells were imaged on a Live Cell Imaging System (BioTek, Lionheart FX) or a Leica SP5 laser scanning confocal microscope.

### Proteomics and bioinformatics analysis

Fresh liver tissues were shipped to Shanghai Applied Protein Technology for TMT-graded proteomics analysis. Samples were subjected to protein extraction using 4% SDS, trypsin digestion, TMT labeling, and liquid chromatography–mass spectrometry. Differentially expressed proteins were screened according to the expression ratio change of more than 1.2 times (up-regulated greater than 1.2 times or down-regulated less than 0.83 times) and *P* value < 0.05 [23].

Plasma was collected from fasted mice and shipped to Shenzhen BGI Co. Ltd. for proteomics analysis. Samples were subjected to protein extraction with free SDS lysate, quality control, trypsinolysis, high pH RP separation, Data Dependent Acquisition database construction, and Data Independent Acquisition quantitative detection by liquid chromatography–mass spectrometry [23]. Using the MSstats software package, the error correction and normalization of each sample were completed. The differences between different proteins were then assessed according to the set of comparison groups and the linear mixed-effects model. Fold change >2 and *P* value <0.05 were used as screening criteria for significantly different proteins. The differential proteins were analyzed using Euclidean distance and the hierarchical cluster method.

Proteins were applied to GO functional annotations and function enrichment analysis using Blast2Go (https://www.blast2go.com/) and Fisher’s exact test method [23]. The significance level of the protein enrichment pathway was analyzed based on the KEGG database and calculated using Fisher's exact test.

### AAV-shRNA preparation and administration

Recombinant adeno-associated virus (AAV) 8 were generated by transfecting 293T HEK cells with AAV8 vectors containing shRNA SAA1 (F: GAAGGAAGCUAACUGGAAATT; R: UUUCCAGUUAGCUUCCUUCTT; Loop: CTCGA) under the control of the mouse U6 promoter. Cell lysate was subjected to freeze/thaw and treated with 50 U/ml Benzonase for 30 min at 37°C and clarified by centrifugation. AAV8 were purified using iodixanol gradient ultracentrifugation, followed by dialysis against Dulbecco’s phosphate-buffered saline using centrifugal filters [67]. AAV particles were titered as viral genomes per ml (vg/ml) using qRT-PCR. Each mouse was injected with 1 × 10^11^ vg intravenously.

### Statistical analysis

Statistical analysis was conducted using Prism 9 (Version10.2.3). The significance between 2 groups was assessed using Student’s *t* test (unpaired, 2-tailed). One-way or 2-way ANOVA (post-hoc test, Bonferroni or Tukey) was used to evaluate the significance among multiple groups. Data were presented as mean ± SD of at least 3 biological replicates per group. *P* < 0.05 was set as statistically significant.

## Data Availability

All supporting data are available within the article and its online Supplementary Materials. Additional data may be provided by the corresponding authors upon reasonable request.
