# Supplementary material for: Hepatic Surf4 Deficiency Impairs Serum Amyloid A1 Secretion and Attenuates Liver Fibrosis in Mice
Source: Research (Wash D C). 2024 Aug 5;7:0435. doi: 10.34133/research.0435 (PMC11298252; doi:10.34133/research.0435)
Supplement: Supplementary 1 — Figs. S1 to S9 Tables S1 and S2 [file research.0435.f1.zip › Supply total-Research.pdf]

Hepatic Surf4 Deficiency Impairs SAA1 Secretion and Attenuates Liver Fibrosis in Mice

Bingxiang Wang[1,2,#,*], Huili Li[1,2,#], Govind Gill[3], Xiangyan Zhang[4], Geru Tao[1,2], Boyan Liu[1,2], Lei Zhai[1,2], Wei Chen[1,2], Hao Wang[1,2], Hong-mei Gu[3], Shucun Qin[1,2,*], Da-wei Zhang[3,*]

Short title: Surf4 and liver fibrosis

[*]**Address correspondence to**: Bingxiang Wang; wangbingxiang@sdfmu.edu.cn, Shucun Qin; scqin@sdfmu.edu.cn, and Dawei Zhang; dzhang@ualberta.ca

# These authors contributed equally to this work.

Supplementary Materials

Fig. S1. GO function enrichment analysis of liver protein.
Fig. S2. Plasma lipid levels of CCl_4-treated mice.
Fig. S3. Blood glucose and body and liver weight.
Fig. S4. Impact of Surf4 deficiency on liver fibrosis.
Fig. S5. Hepatic TGF-β expression and plasma proteins.
Fig. S6. Proteomics analysis of up- and down-regulated plasma proteins.
Fig. S7. The expression and location of SAA1 in primary hepatocytes.
Fig. S8. Effect of SAA1 on HSCs activation.
Fig. S9. Analysis of human liver samples and a proposed model.
Table S3 Primers
Table S4 List of Antibody

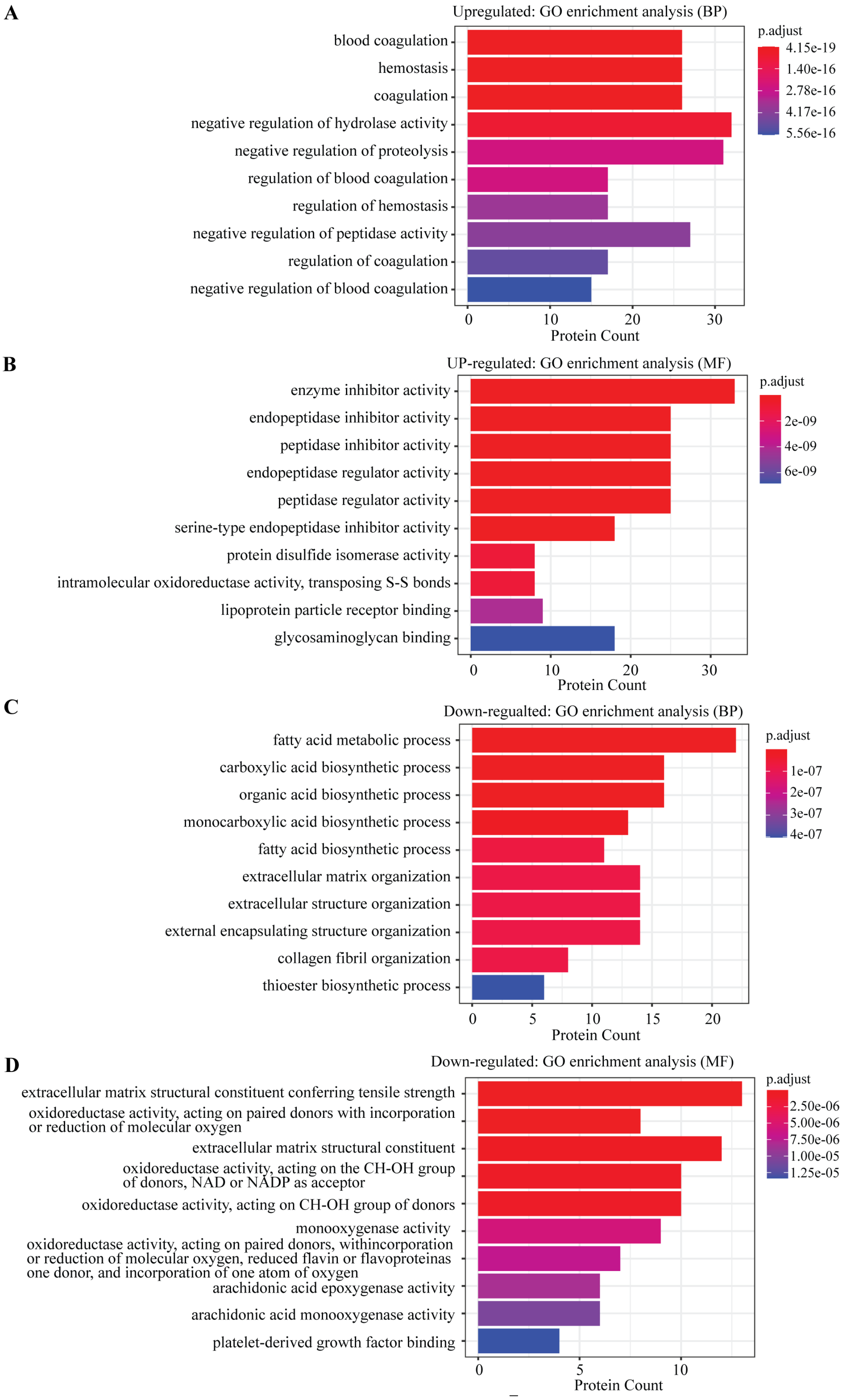
A
Upregulated: GO enrichment analysis (BP)
blood coagulation
hemostasis
coagulation
negative regulation of hydrolase activity
negative regulation of proteolysis
regulation of blood coagulation
regulation of hemostasis
negative regulation of peptidase activity
regulation of coagulation
negative regulation of blood coagulation
0 10 20 30
Protein Count
p.adjust
4.15e-19
1.40e-16
2.78e-16
4.17e-16
5.56e-16
B
UP-regulated: GO enrichment analysis (MF)
enzyme inhibitor activity
endopeptidase inhibitor activity
peptidase inhibitor activity
endopeptidase regulator activity
peptidase regulator activity
serine-type endopeptidase inhibitor activity
protein disulfide isomerase activity
intramolecular oxidoreductase activity, transposing S-S bonds
lipoprotein particle receptor binding
glycosaminoglycan binding
0 10 20 30
Protein Count
p.adjust
2e-09
4e-09
6e-09
C
Down-regualted: GO enrichment analysis (BP)
fatty acid metabolic process
carboxylic acid biosynthetic process
organic acid biosynthetic process
monocarboxylic acid biosynthetic process
fatty acid biosynthetic process
extracellular matrix organization
extracellular structure organization
external encapsulating structure organization
collagen fibril organization
thioester biosynthetic process
0 5 10 15 20
Protein Count
p.adjust
1e-07
2e-07
3e-07
4e-07
D
Down-regulated: GO enrichment analysis (MF)
extracellular matrix structural constituent conferring tensile strength
oxidoreductase activity, acting on paired donors with incorporation or reduction of molecular oxygen
extracellular matrix structural constituent
oxidoreductase activity, acting on the CH-OH group of donors, NAD or NADP as acceptor
oxidoreductase activity, acting on CH-OH group of donors
monooxygenase activity
oxidoreductase activity, acting on paired donors, withincorporation or reduction of molecular oxygen, reduced flavin or flavoproteinas one donor, and incorporation of one atom of oxygen
arachidonic acid epoxygenase activity
arachidonic acid monooxygenase activity
platelet-derived growth factor binding
0 5 10
Protein Count
p.adjust
2.50e-06
5.00e-06
7.50e-06
1.00e-05
1.25e-05

Fig. S1. GO function enrichment analysis of liver protein. (A and B) Upregulated protein (n=4 mice). (C and D) Downregulated proteins (n=4 mice). Data were analyzed using Fisher's exact test. Graphics were generated by the Hiplot Pro Biomedical visualization platform of Shanghai Tengyun cloud mapping system.

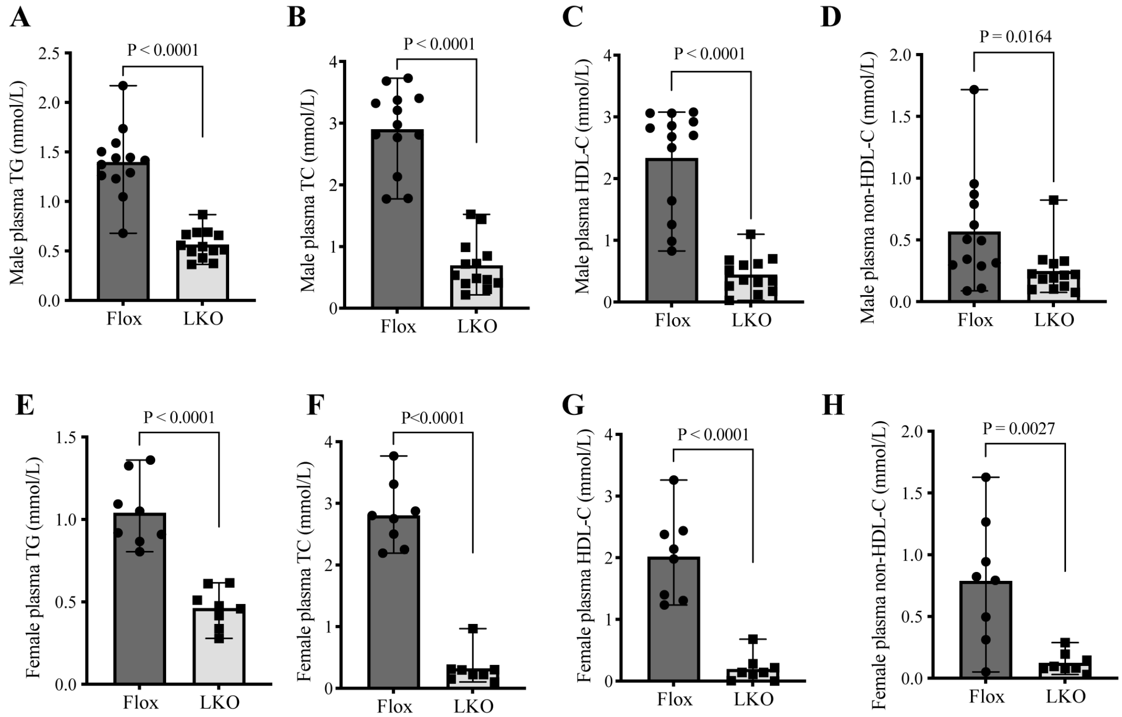

Fig. S2. Plasma lipid levels of CCl_4-treated mice. Male and female *Surf4*^{flox} and *Surf4*^{LKO} mice (10-12 weeks old, regular diet, n=8-13 mice) were injected with CCl_4 for 6 weeks and fasted for 10 h before blood collection. (A and E) Plasma triglycerides (TG). (B and F) Total cholesterol (TC). (C and G) Plasma HDL-C. (D and H) Plasma non-HDL-C. Statistics: Unpaired Student's *t*-test.

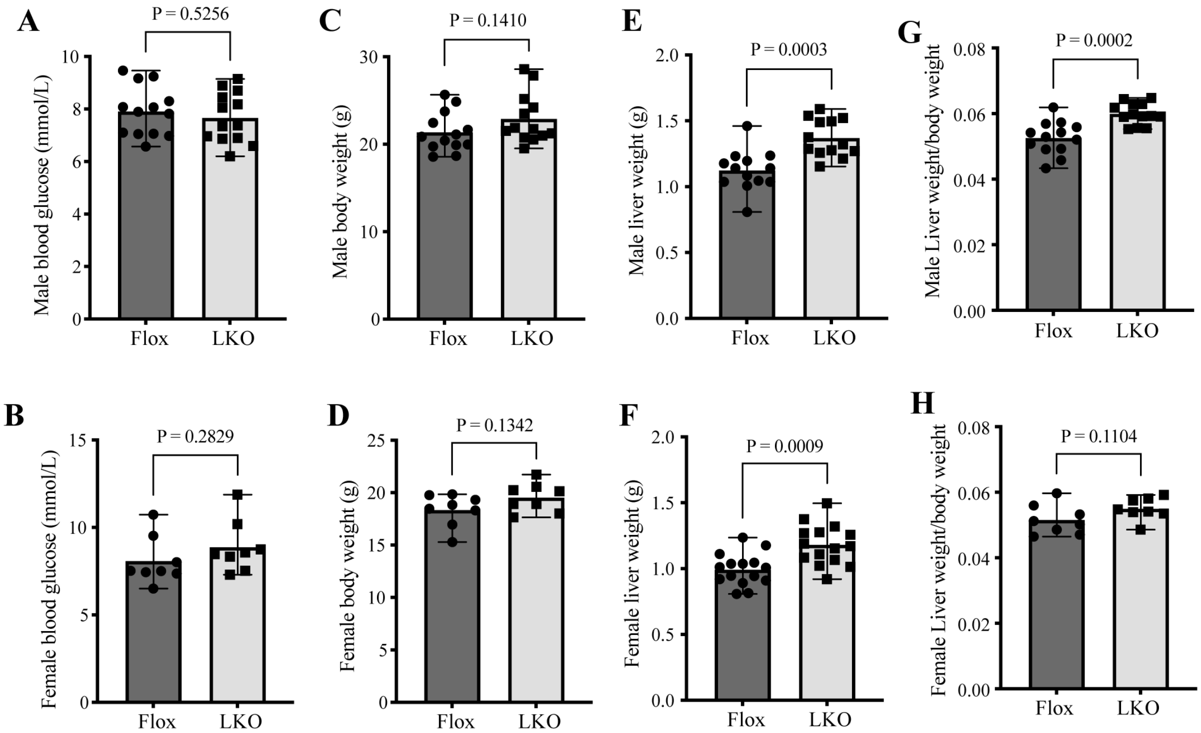

Fig. S3. Blood glucose and body and liver weight. Male and female mice were treated as described in Fig. S2 legend. (A and C) Plasma glucose levels. (B and D) Body weight. (E and F) Liver weight. (G and H) Liver/body weight ratio (13-15 mice per group).

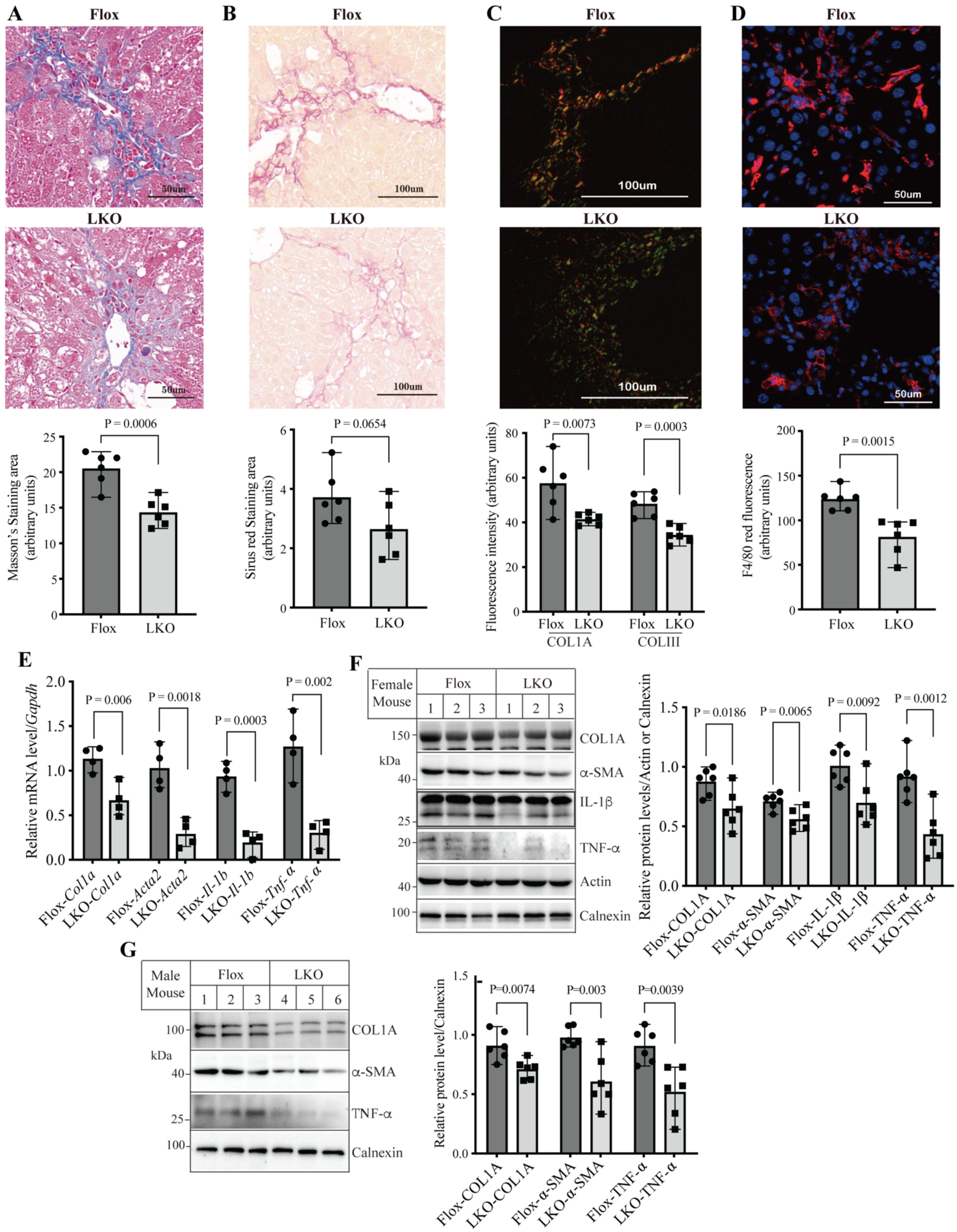

Fig. S4. Impact of Surf4 deficiency on liver fibrosis. (A to C) Histology of liver sections from female mice treated as in Figure S2 legend. Masson's (A) or Sirius red staining (B) followed by polarized light

microscopy (C) to quantify collagen content (red and green indicated COLI and III, respectively) (n=6 mice). (D) Immunostaining of female liver sections with F4/80 (Red, n=6 mice). (E) qRT-PCR (n=4 mice). The mRNA level of target genes was normalized to that of *Gapdh* for relative mRNA levels. (F) Immunoblotting of equal amount of male and female liver homogenate (n=6 mice). Relative levels were calculated by normalizing the densitometry of COL1A, IL-1β, or TNF-α to that of Actin or the densitometry of α-SMA to that of Calnexin on the same blot. (G) Immunoblotting of whole cell lysate of primary HSCs isolated from male $Surf4^{flox}$ or $Surf4^{LKO}$ mice (n=6 mice). Relative expression was the normalization of the densitometry of targets to that of Calnexin on the same blot. Images in panels A-D and F-G were representative. Data were quantified using Image J.

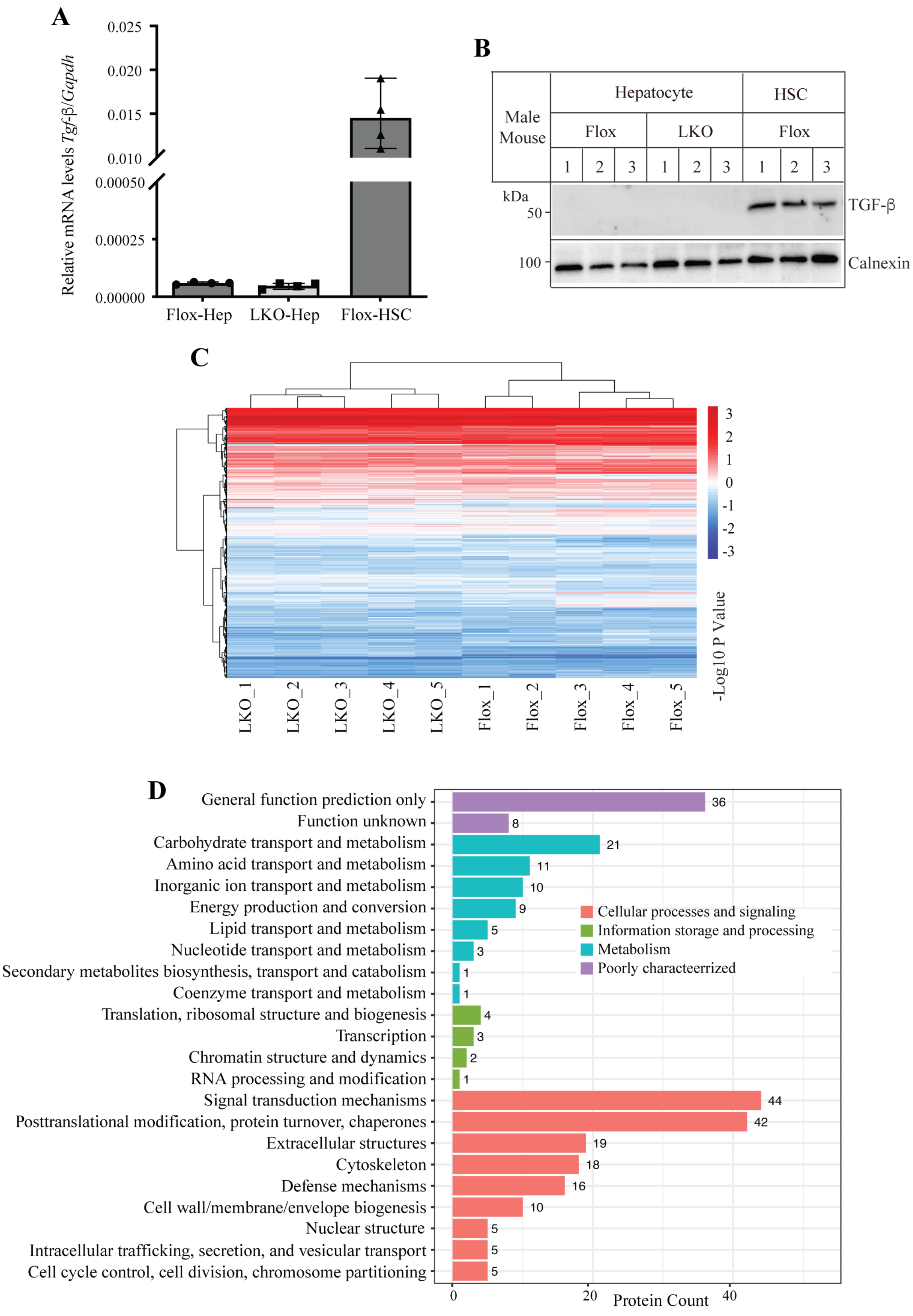

Fig. S5. Hepatic TGF-β expression and plasma proteins. Male *Surf4*^{flox} and *Surf4*^{LKO} mice (10-12 weeks old) were fed a regular diet. (A) qRT-PCR of total RNA from hepatocytes and HSCs (n=4 mice). The mRNA level of *Tgf-β* was normalized to that of *Gapdh* for relative levels. (B) Immunoblotting of

the same amount of cell lysate (n=3 mice). (C and D) Proteomics of plasma of different male mice (n=5), including heat map (C), functional classification statistical analysis of differential proteins based on Eukaryotic orthologous groups (KOGs) (D). Data were analyzed as described in Fig. S1 legend. D: The ordinate is the KOG classification entry, and the horizontal axis is the number of proteins corresponding to the functional classification.

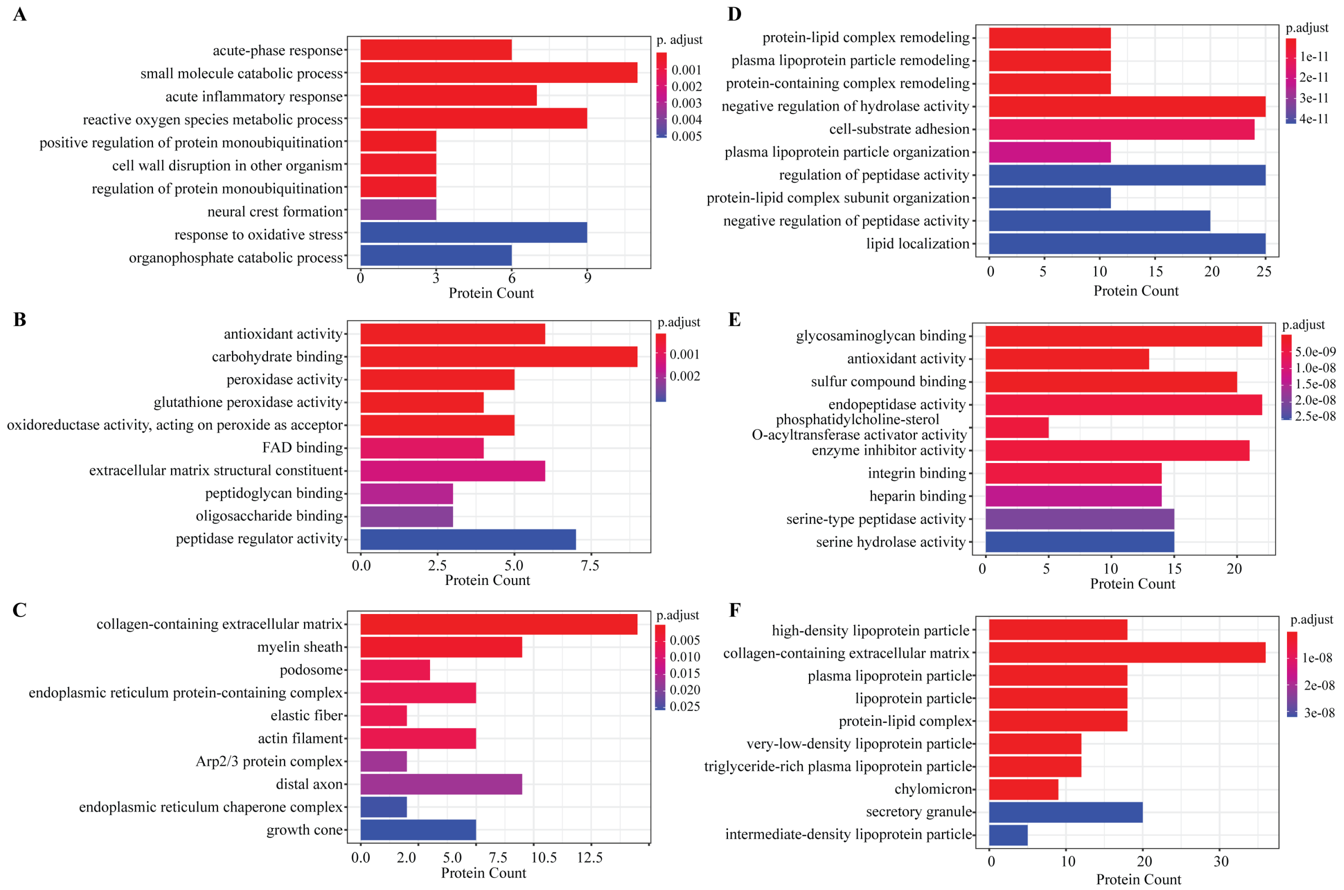

Fig. S6. Proteomics analysis of up- and down-regulated plasma proteins. Fasting plasma of male *Surf4*^{flox} and *Surf4*^{LKO} mice was collected for proteomics (n=5 mice, regular diet). (A-C) GO function analysis of up-regulated proteins. (D-F) GO function analysis of down-regulated proteins. (A and D) Biological Process. (B and E) Molecular Function. (C and F) Cellular Component. Graphics were generated using the Hiplot Pro Biomedical visualization platform of the Shanghai Tengyun cloud mapping system.

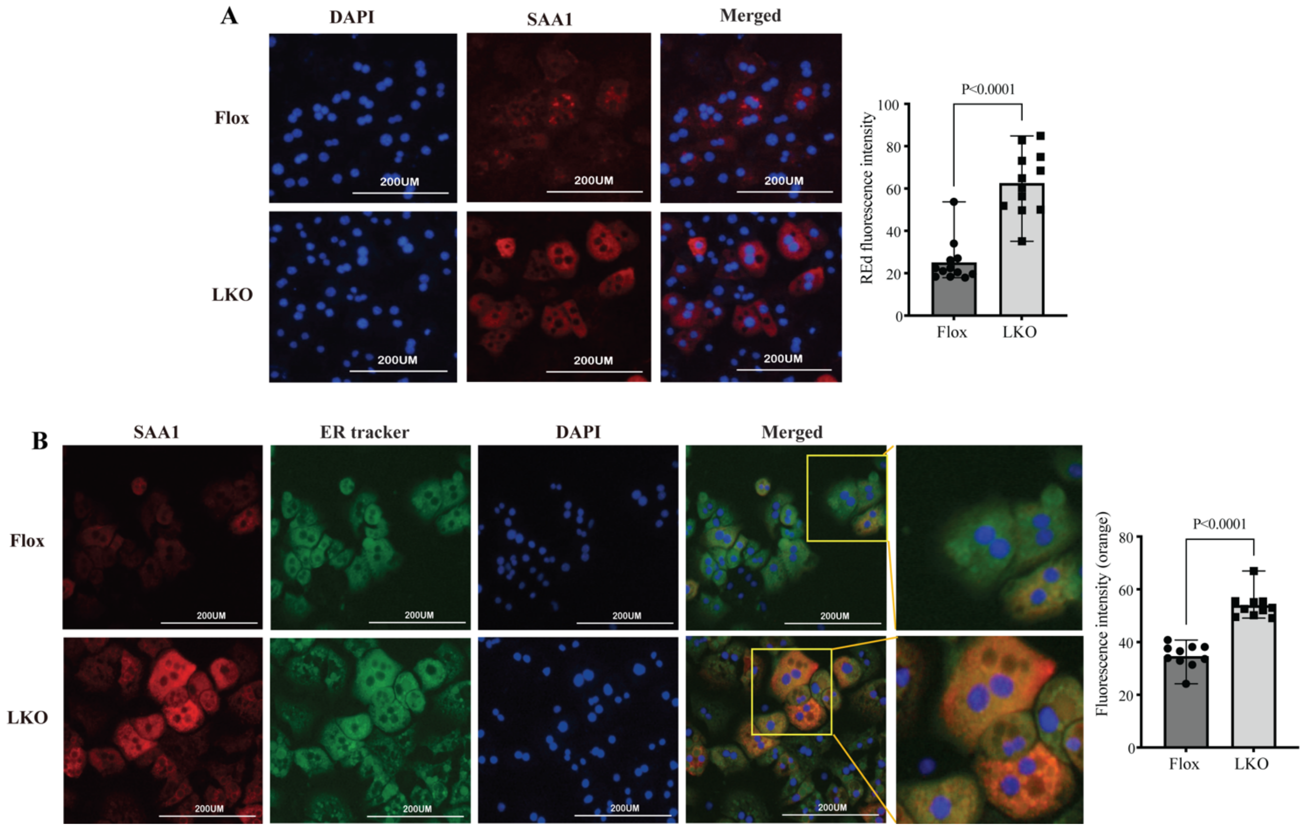

Fig. S7. The expression and location of SAA1 in primary hepatocytes. (A and B) Immunofluorescence. Primary hepatocytes were isolated from male *Surf4*^{flox} and *Surf4*^{LKO} mice (10-12 weeks old) fed a regular chow diet. SAA1 expression in primary hepatocytes (A, n=12 biological replicates) and colocalization of SAA1 and ER in primary hepatocytes (B, n=10 biological replicates). SAA1, red. Nuclei (DAPI): Blue. ER: Green. Representative images were shown. SAA1 staining (A) and co-staining of SAA1 with an ER tracker (B) were quantified using Image J.

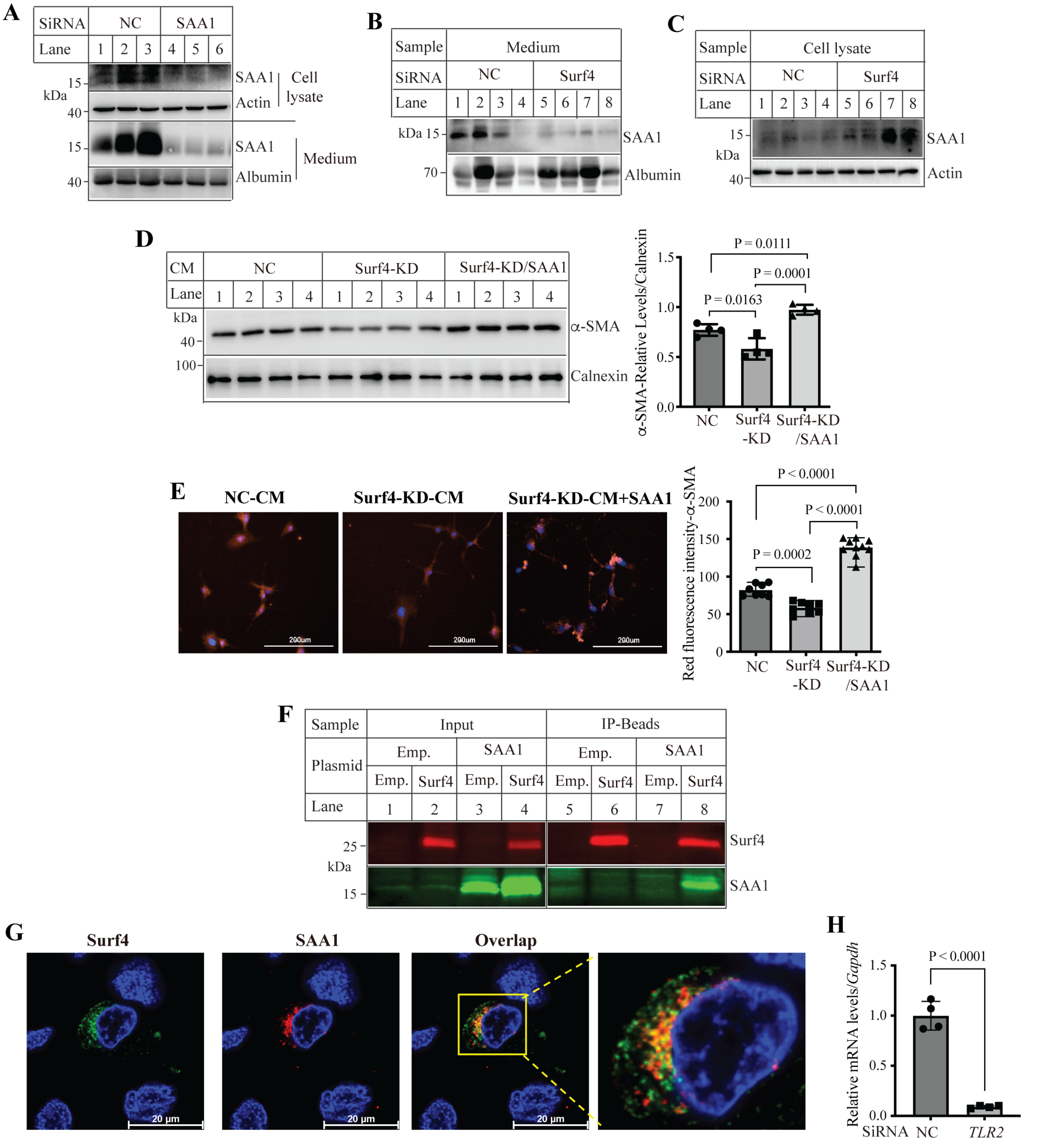

Fig. S8. Effect of SAA1 on HSCs activation. (A) Knockdown of SAA1 in primary hepatocyte of different mice receiving CCl_4. Primary hepatocytes from mice on a regular diet administered CCl_4 (n=3 mice) were transfected with NC or SAA1 siRNA. 24h later, cells were cultured in medium without FBS (conditioned medium/CM). 24 h after, medium and cell lysate were prepared for immunoblotting. (B and C) Surf4 knockdown. 24 h after transfection with negative control (NC) or SAA1 siRNA, HepG2 cells were washed and cultured in medium without FBS. 24 h later, CM and cell lysate were isolated for Western Blot to detect SAA1 in culture medium and cell lysate. (D and E) Effects of CM from Surf4

knockdown HepG2 cells on LX-2 cells. HepG2 cells were transfected with NC or Surf4 siRNA. 48h later, CM was harvested to treat LX-2 cells with or without recombine SAA1. α-SMA was monitored by immunoblotting (D, n=4 biological replicates) and immunofluorescence (E, red). Relative expression was calculated by normalizing α-SMA densitometry to Calnexin densitometry on the same blot. Statistic: One-way ANOVA. (F) Immunoprecipitation. HepG2 cells were transfected with the same amount of empty, Surf4+Empty, SAA1+Empty, or Surf4+SAA1 plasmids. An equal amount of cell lysate was used to immunoprecipitate Myc-tagged Surf4 using anti-Myc-conjugated beads. Immunoprecipitated proteins were monitored by immunoblotting (n=3 biological replicates). (G) Confocal microscopy. Surf4 and SAA1 were transiently co-transfected into HepG2 cells. Distribution of SAA1(red) and Surf4 (green) was detected by confocal microscopy. DAPI: Blue. (H) Knockdown of TLR2 in LX-2 cells. 48 h after transfection with NC or TLR2 siRNA, LX-2 cells were collected. qRT-PCR was conducted to measure *TLR2* mRNA. Representative images were shown. Data were quantified using Image J .

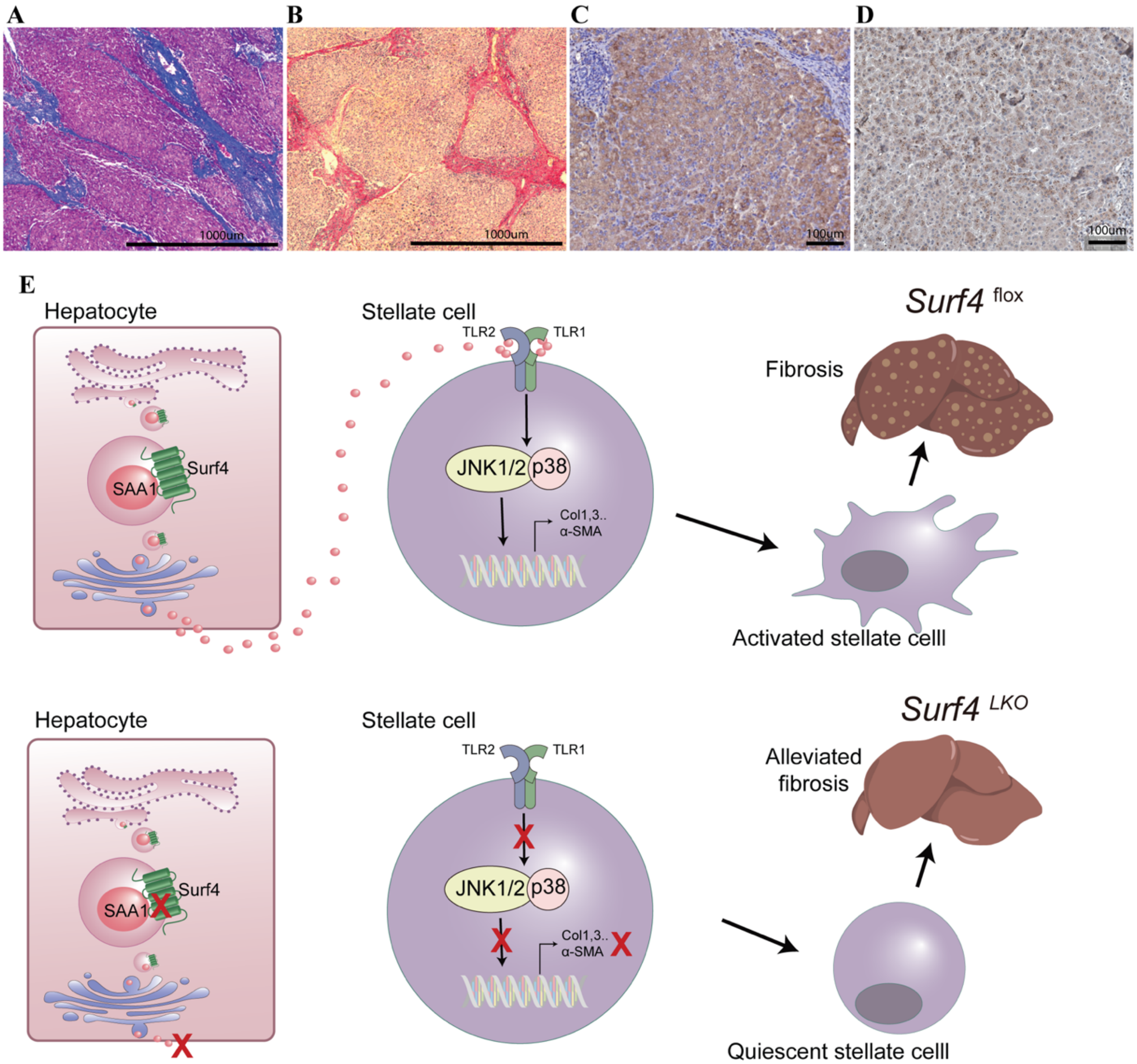

Fig. S9. Analysis of human liver samples and a proposed model. (A and B) Staining of human liver section (n=15 patient samples). A, Masson's staining. B, Sirius red staining. (C and D) IHC of human liver samples with Surf4 (n=50 patient samples) and SAA1 (n=20 patient samples). Representative images were shown. (E) A working model showing that the Surf4-SAA1-TLR2 pathway may mediate communication between hepatocytes and HSCs.

Table S1 Primers.

Genotyping

Name	Primer sequence
Surf4 5’ Loxp	F: 5′-AATGCTGCTTGTGGCATCTCAAAGG-3′<br>R: 5′-CTACCAGGT TTGTTCCACCCTCCAA-3′
Surf4 3’ Loxp	F: 5′-CCAGAAAGGACAAAAGGGTTCAGTC-3′<br>R: 5′-TACAAGGCCTTTCTCACCTCCTAA C-3′
Alb-Cre	20239: 5′-TGCAAACATCACATGCACAC-3′<br>20240: 5′-TTGGCCCCTTACCATAACTG-3′<br>5374: 5′-GAAGCAGAAGCTTAGGAAGATGG-3′

qRT-PCR Primers

Name	Primer sequence
Mouse *Gapdh*	F: 5′-AGGTCGGTGTGAACGGATTTG-3′<br>R: 5′-TGTAGACCATGTAGTTGAGGTCA-3′
Mouse *α-SMA*<br>（*ACTA2*）	F: 5′-GTCCCAGACATCAGGGAGTAA-3′<br>R: 5′-TCGGATACTTCAGCGTCAGGA-3′
Mouse *Col1α*	F: 5′- GCTCCTCTTAGGGGCCACT-3′<br>R: 5′- CCACGTCTCACCATTGGGG-3′
Mouse *Tnfα*	F: 5′- CCCTCACACTCAGATCATCTTCT-3′<br>R: 5′- GCTACGACGTGGGCTACAG-3′
Mouse *Il-1β*	F: 5′- GCAACTGTTCCTGAACTCAACT-3′<br>R: 5′- ATCTTTTGGGGTCCGTCAACT-3′
Mouse *Surf4*	F: 5′- CCTCCTTCACTTTGATGCCAG-3′<br>R: 5′- AAGGCGTTGAAATACACGTT-3′
Mouse *Ltbp1*	F: 5′- CCAGTCCCAAGTCTCTTACCA-3′<br>R: 5′- CTGGAAGCATCGGCCAAGT-3′
Mouse *Tgfβ1*	F: 5′- CTCCCGTGGCTTCTAGTGC-3′<br>R: 5′- GCCTTAGTTTGGACAGGATCTG-3′
Human *α-SMA*<br>（*ACTA2*）	F: 5′- AAAAGACAGCTACGTGGGTGA-3′<br>R: 5′- GCCATGTTCTATCGGGTACTTC-3′
Human *COL1α*	F: 5′- GAGGGCCAAGACGAAGACATC-3′

	R: 5′- CAGATCACGTCATCGCACAAC-3′
Human *TGFβ1*	F: 5′- CTAATGGTGGAAACCCACAACG-3′<br>R: 5′- TATCGCCAGGAATTGTTGCTG-3′
Human *TLR2*	F: 5′- TTATCCAGCACACGAATACACAG-3′<br>R: 5′- AGGCATCTGGTAGAGTCATCAA-3′

siRNA sequence

Name	Primer sequence	Cat. No.	Company
Surf4 (human) siRNA-159	F: 5′-CUUAAAGGAACCAAUGAGUTT-3′<br>R: 5′-ACUCAUUGGUUCCUUUAAGTT-3′		Reach Cloud Biology
Negative Control siRNA	F: 5′- UUCUCCGAACGUGUCACGUTT-3′<br>R: 5′- ACGUGACACGUUCGGAGAATT-3′		Reach Cloud Biology
TLR2 (human) siRNA		sc-40256	Santa Cruz
Saa1 (mouse) siRNA-159	F: 5′-GAAGGAAGCUAACUGGAAATT-3′<br>R: 5′-UUUCCAGUUAGCUUCCUUCTT-3′		Reach Cloud Biology

Table S2. Antibody list.

Name	Cat. No.	Lot. No.	Company	Notes
TNF α	60291-1-IG	10026111	Proteintech	WB
IL-1β	26048-1-AP	00103356	Proteintech	WB
α-SMA	14395-1-AP	00120136	Proteintech	IF
α-SMA	bs-10196R	BC04208443	Bioss	WB
COLIA	bs-0578R	BB05242657	Bioss	WB
F4/80	ab-300421	1043287-38	Abcam	IF
SAA1 + SAA2	ab199030/ab207445	1002190-3/1028482-1	abcam	WB/IF
SAA1	bs-19359R	BC04629913	Bioss	IHC
Phospho-p38MAPK (Thr180/Tyr182)	9211s	21	CST	WB
p38 MAPK	14064-1-AP	00115026	Proteintech	WB

Phospho-SAPK/JNK (Thr183/Tyr185)	9251s	21	CST	WB
JNK	24164-1-AP	00115795	Proteintech	WB
ACTIN	bs0061-R	AH11286402	Bioss	WB
Calnexin	10427-2-AP	00093709	Proteintech	WB
Phospho-JNK (Tyr185)	80024-1-RR	23001681	Proteintech	WB
TLR2	66645-1-IG	10006026	Proteintech	WB
SURF4	11599-1-AP	00076052	Proteintech	WB/IHC
Albumin	16475-1-AP	00078538	Proteintech	WB
LRAT	sc-101391	D2321	Santa Cruz	IF
TGF β	ab215715	1007290-24	Abcam	WB
Myc	600-444-381	22680	Rockland	WB/IP
HA	66006-2-lg	10011878	Proteintech	IF
FLAG	600-441-383	45598	Rockland	IF
