# Supplementary figures and images for: Hepatic Surf4 Deficiency Impairs Serum Amyloid A1 Secretion and Attenuates Liver Fibrosis in Mice
Source: Research (Wash D C). 2024 Aug 5;7:0435. doi: 10.34133/research.0435 (PMC11298252; doi:10.34133/research.0435)

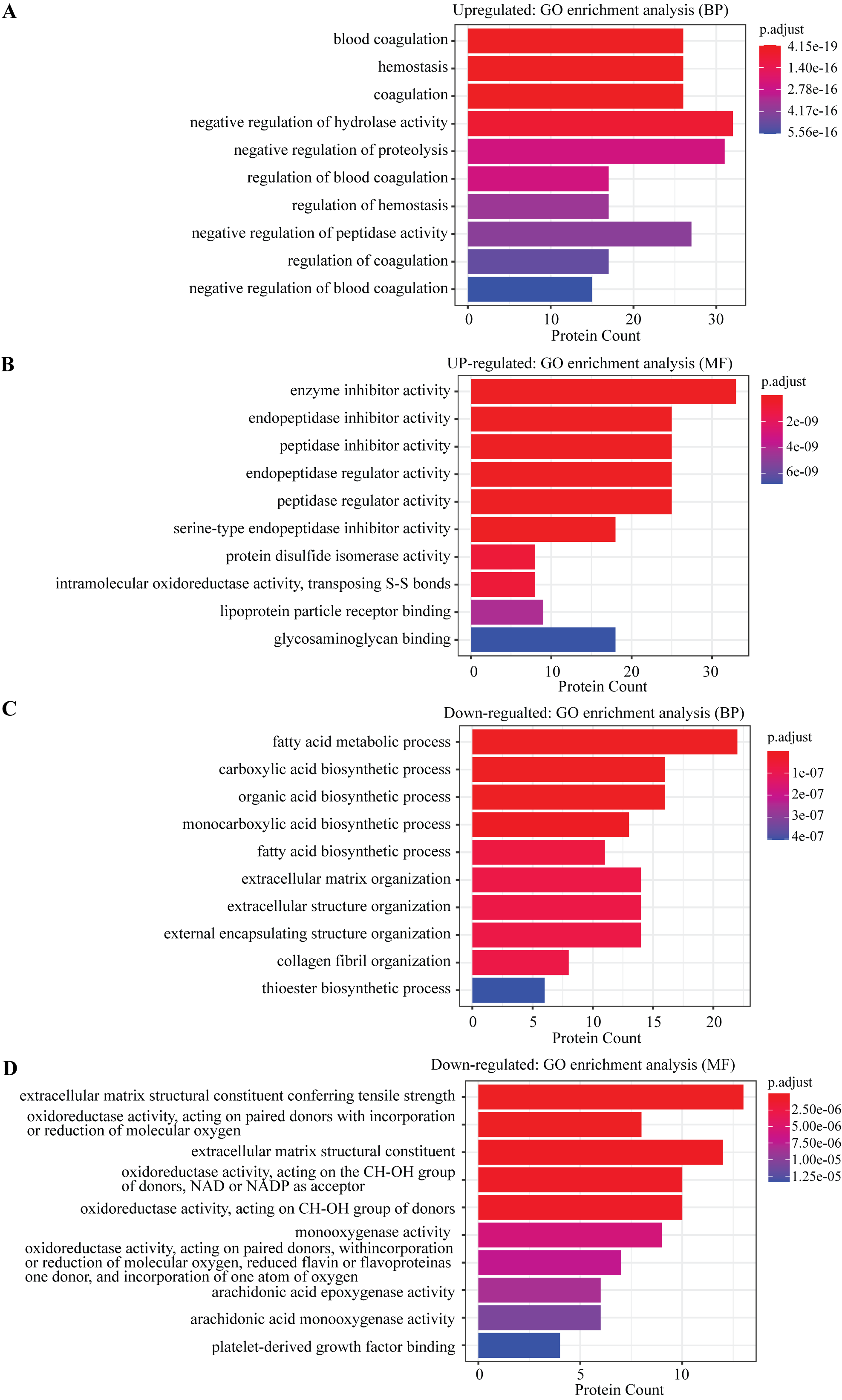

Supplement: Supplementary 1 — Figs. S1 to S9 Tables S1 and S2 [file research.0435.f1.zip › Figure S1-Res.tif]

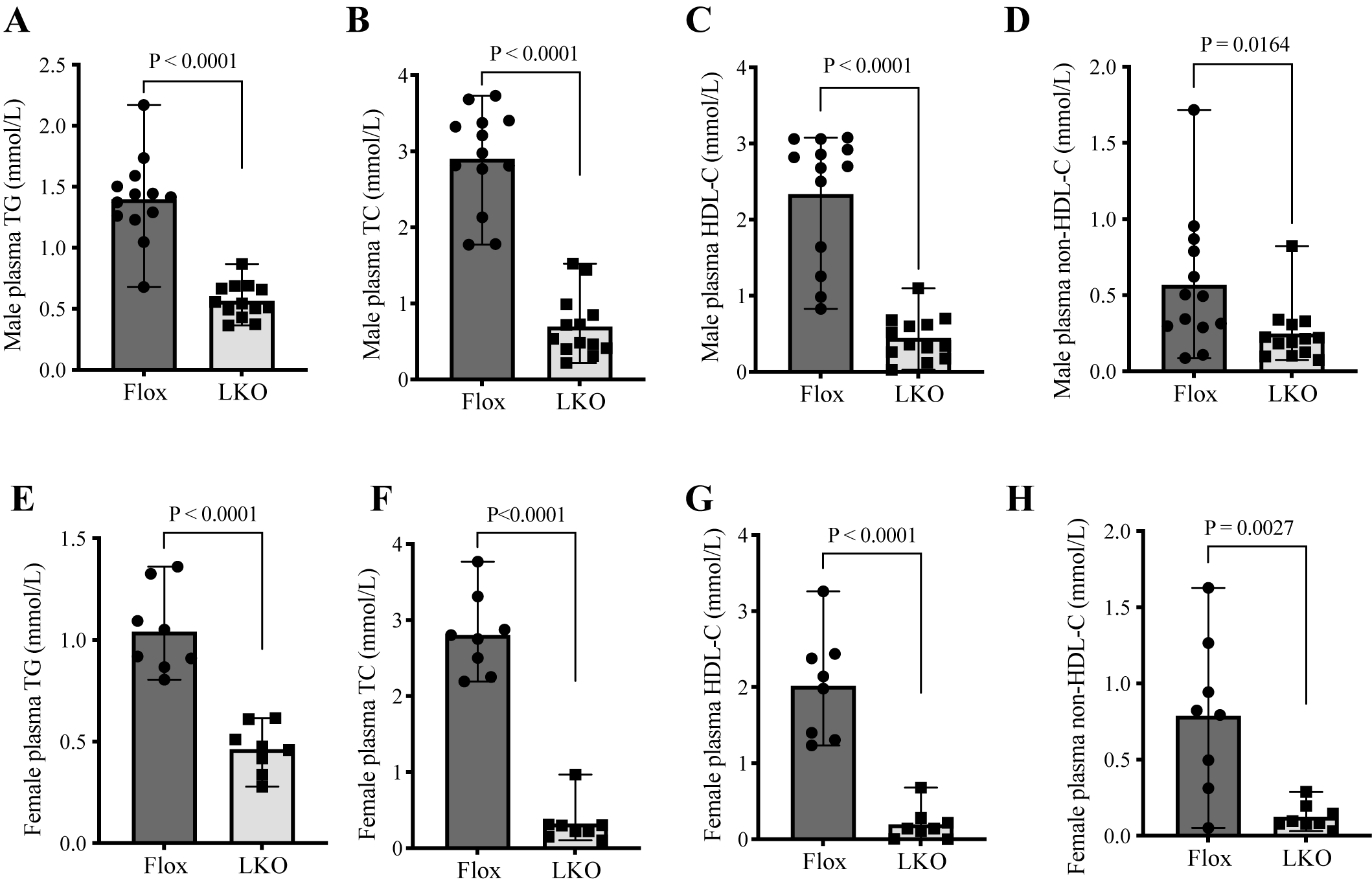

Supplement: Supplementary 1 — Figs. S1 to S9 Tables S1 and S2 [file research.0435.f1.zip › Figure S2-Res.tif]

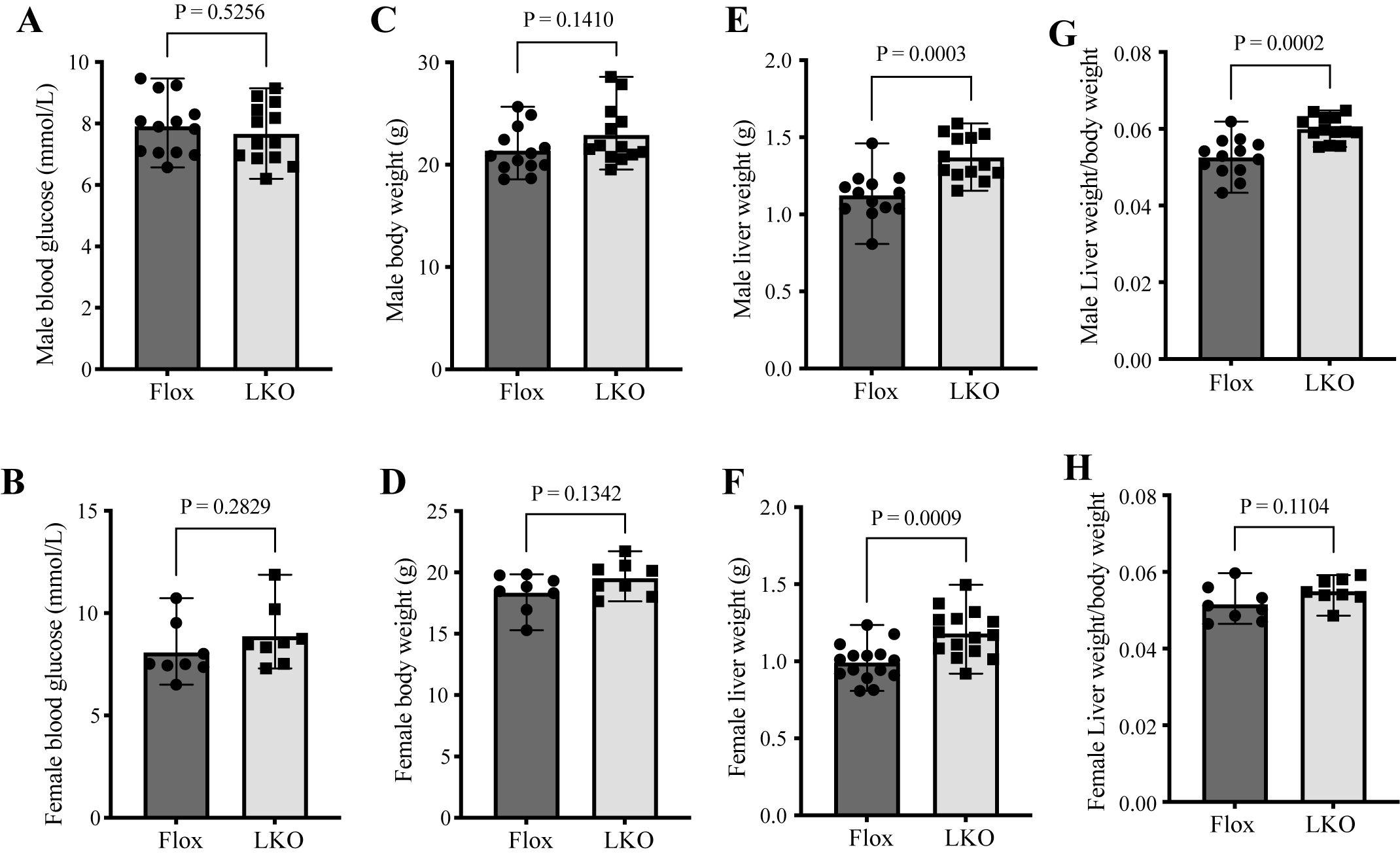

Supplement: Supplementary 1 — Figs. S1 to S9 Tables S1 and S2 [file research.0435.f1.zip › Figure S3-Res.tif]

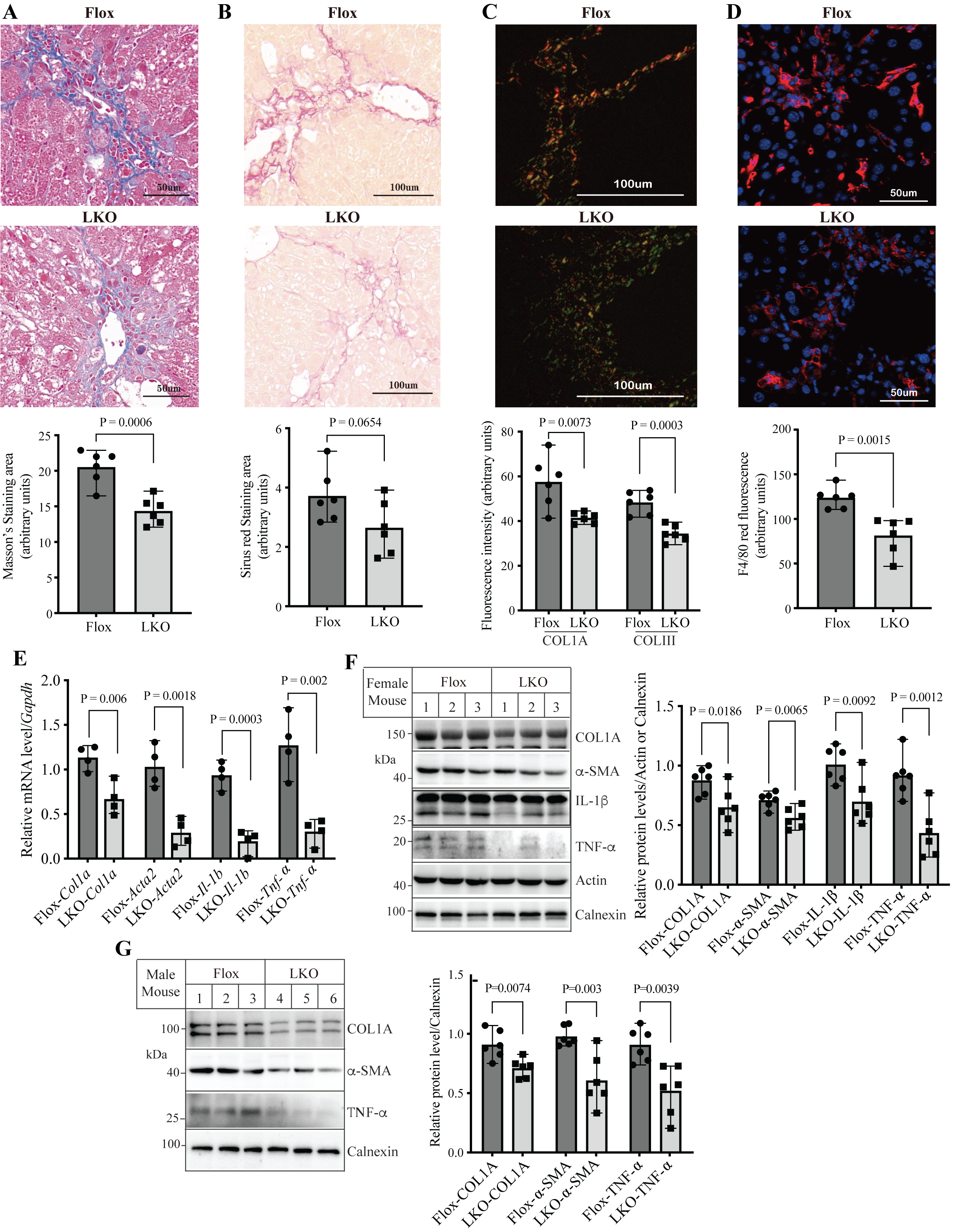

Supplement: Supplementary 1 — Figs. S1 to S9 Tables S1 and S2 [file research.0435.f1.zip › Figure S4-Res.tif]

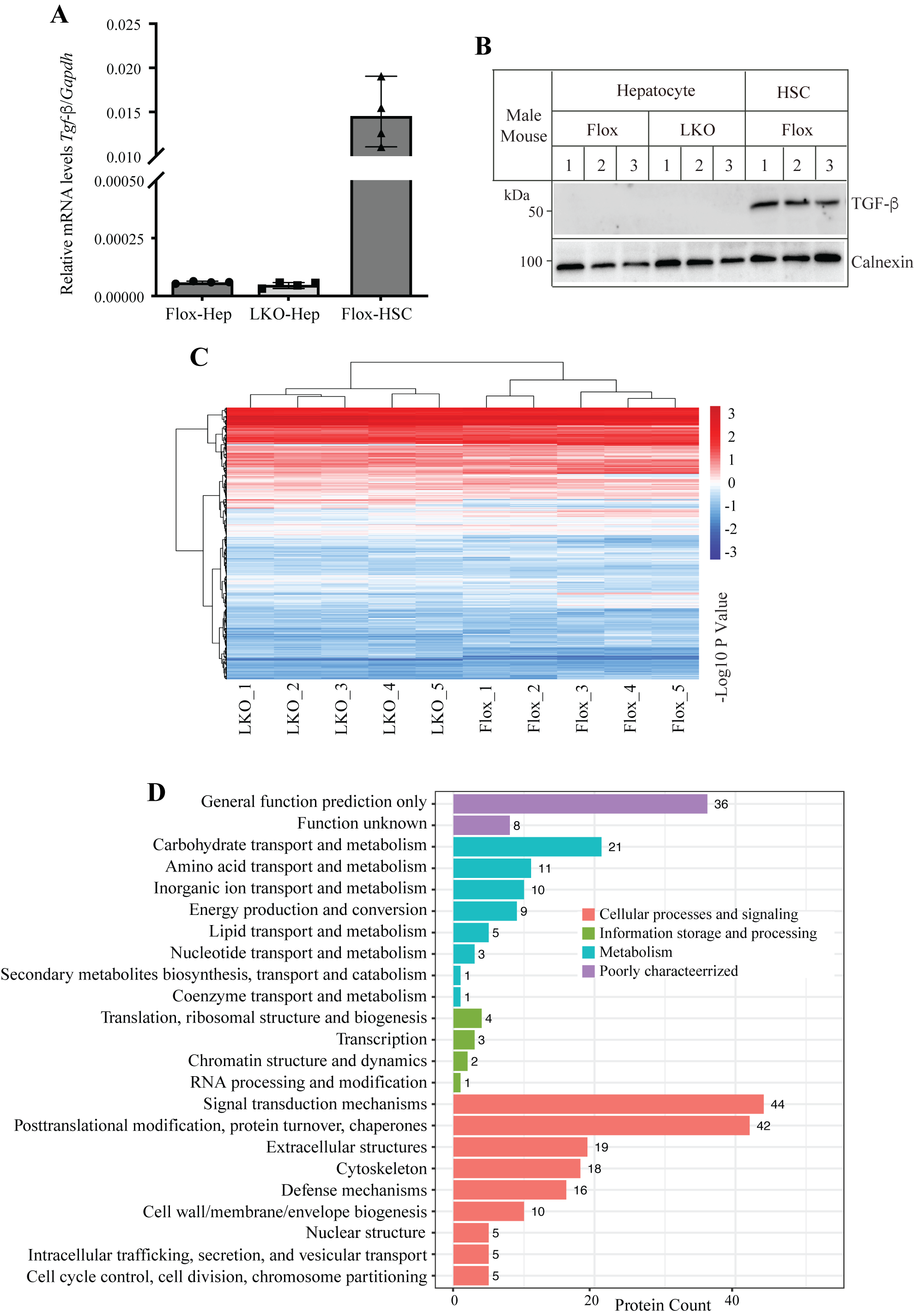

Supplement: Supplementary 1 — Figs. S1 to S9 Tables S1 and S2 [file research.0435.f1.zip › Figure S5-Res.tif]

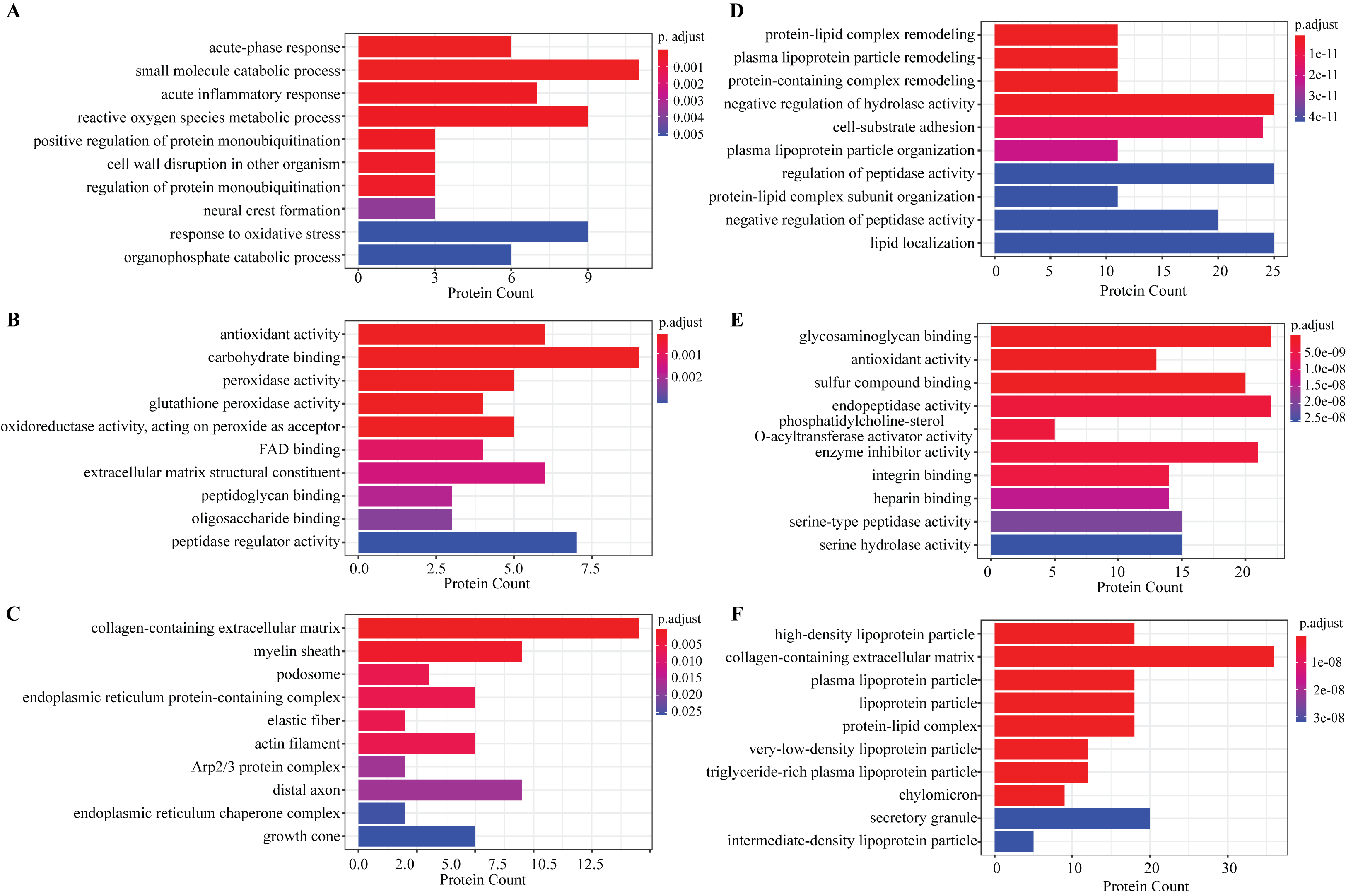

Supplement: Supplementary 1 — Figs. S1 to S9 Tables S1 and S2 [file research.0435.f1.zip › Figure S6-Res.tif]

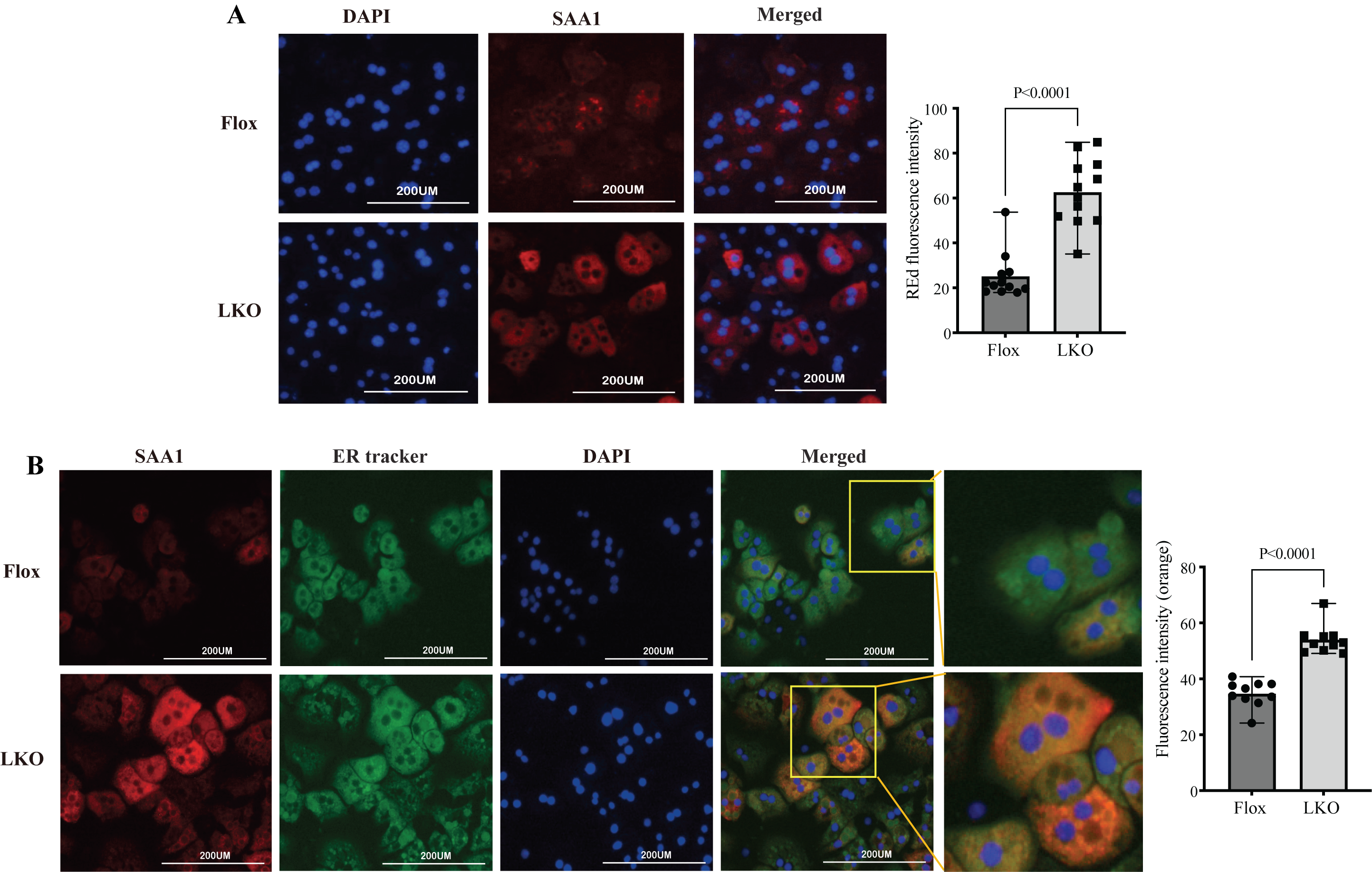

Supplement: Supplementary 1 — Figs. S1 to S9 Tables S1 and S2 [file research.0435.f1.zip › Figure S7-Res.tif]

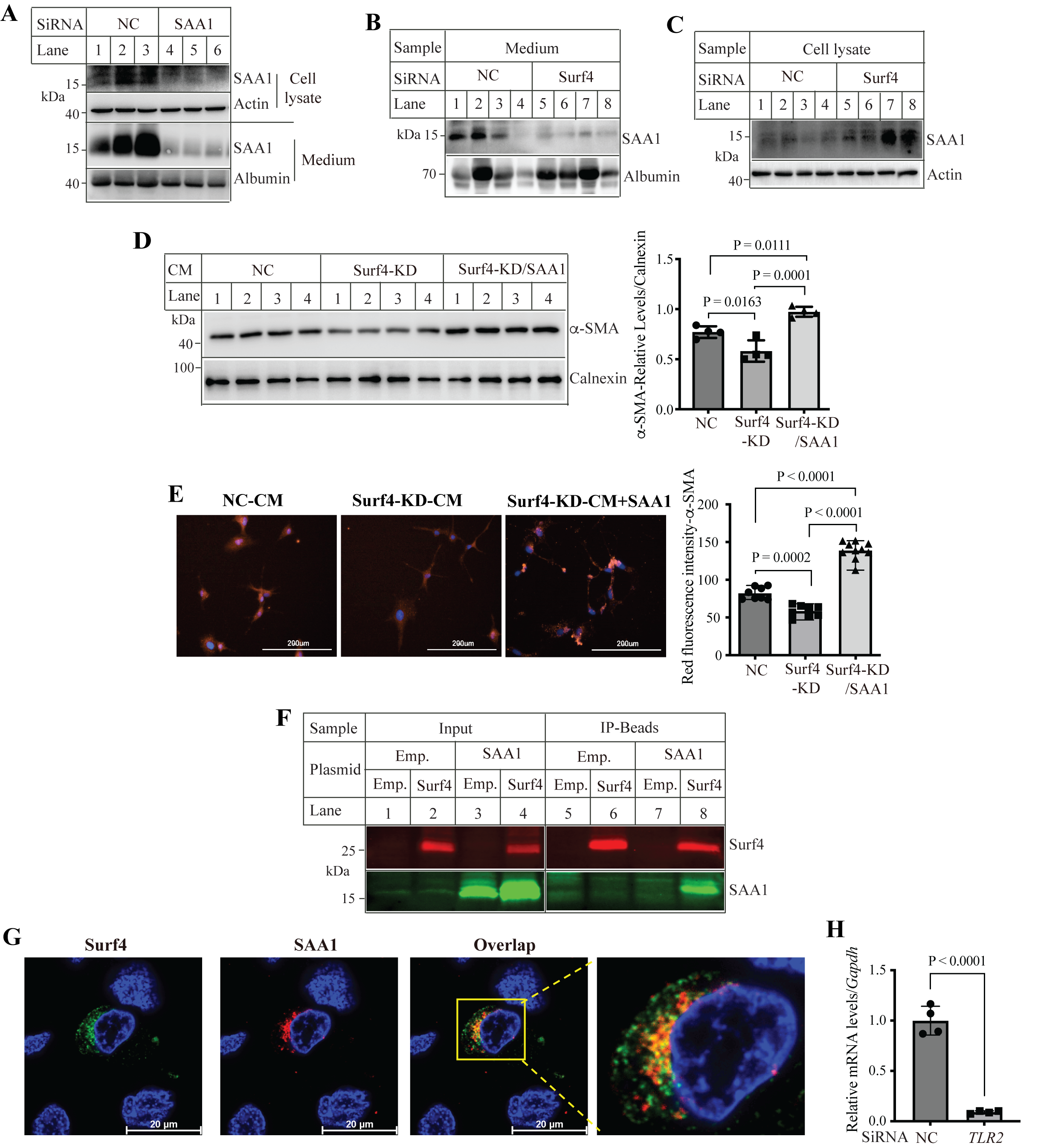

Supplement: Supplementary 1 — Figs. S1 to S9 Tables S1 and S2 [file research.0435.f1.zip › Figure S8-Res.tif]

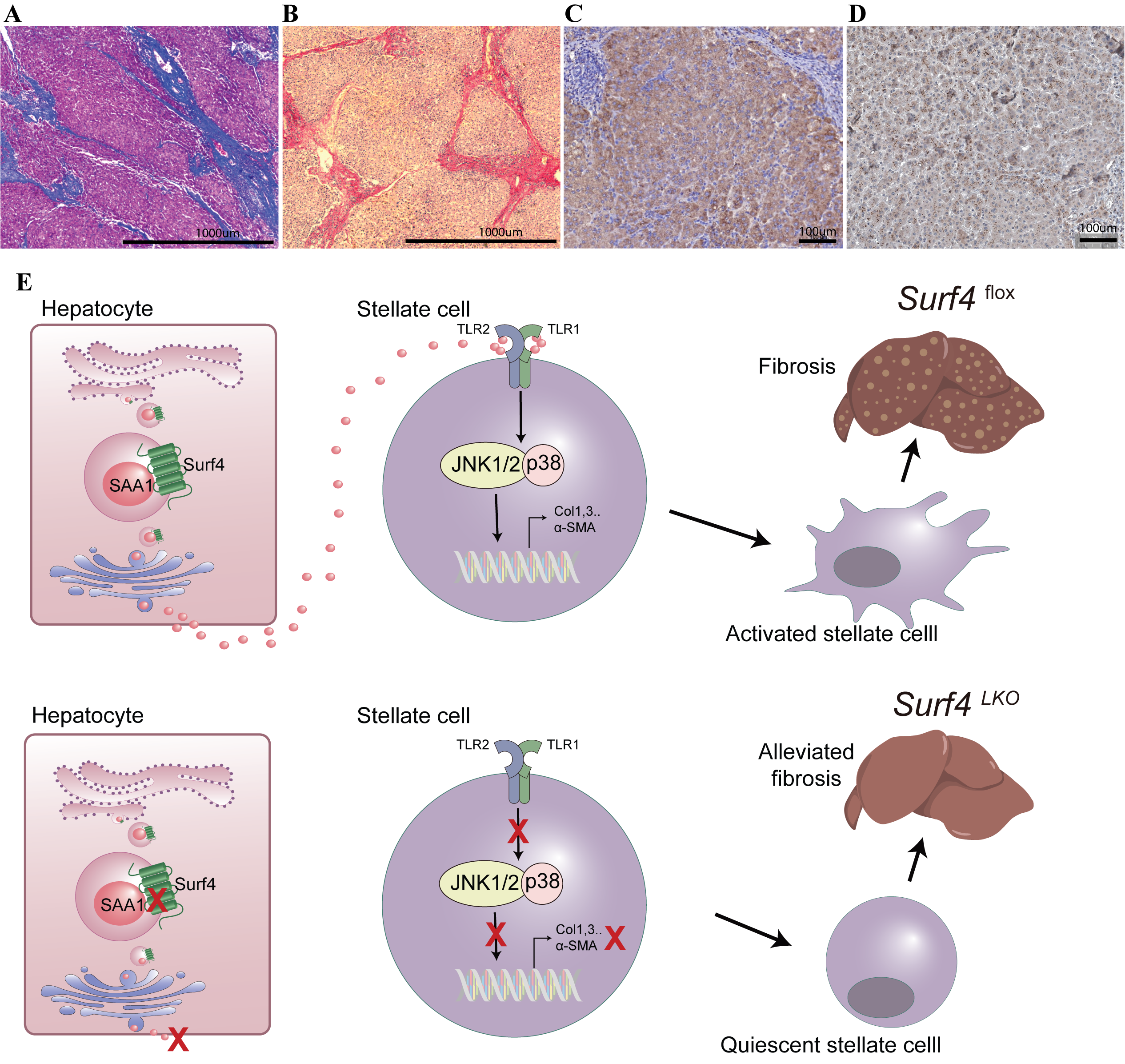

Supplement: Supplementary 1 — Figs. S1 to S9 Tables S1 and S2 [file research.0435.f1.zip › Figure S9-Res.tif]
